# Multi-Objective Optimization of Mechanical Properties for FDM-Printed PLA/TPU Blends via Box–Behnken Design and Entropy Weight Method

**DOI:** 10.3390/polym18172092

**Published:** 2026-08-28

**Authors:** Pei Li, Tianlu Wei, Li Yang, Jing Zhao, Shuo Wang, Shuangjun Wang

**Affiliations:** 1 School of Mechanical and Vehicle Engineering, Bengbu University, Bengbu 233000, China; yang1155@bbc.edu.cn (L.Y.); zhaojingzaozao@163.com (J.Z.); swang2025@bbc.edu.cn (S.W.); 13305522301@163.com (S.W.); 2 Anhui Province Additive Manufacturing Engineering Research Center, Bengbu 233000, China

**Keywords:** fused deposition modeling, PLA/TPU blends, mechanical properties, Box–Behnken design, entropy weight method, multi-objective optimization

## Abstract

Polylactic acid (PLA) is widely used in fused deposition modeling (FDM) due to its excellent mechanical properties and processability. However, its inherent brittleness significantly restricts its application in load-bearing and high-toughness scenarios. To address this limitation, this study establishes a multi-objective optimization framework that integrates single-factor experiments, Box–Behnken design (BBD), response surface methodology (RSM), and entropy weight-based objective weighting to simultaneously enhance the tensile strength, elongation at break, and flexural strength of FDM-printed PLA/thermoplastic polyurethane (TPU) blends. Through single-factor experiments, the optimal PLA/TPU blend ratio was determined as 80:20, achieving a tensile strength of 38.93 MPa, an elongation at break of 14.12%, and a flexural strength of 42.33 MPa—representing improvements of 39.4%, 15.7%, and 31.6%, respectively, over the 70:30 blend. Multi-scale characterization via FTIR, XRD, and SEM reveals that this enhanced performance arises from strong interfacial hydrogen bonding, a well-retained crystalline PLA framework, and the uniform dispersion of fine TPU domains. Subsequently, a four-factor, three-level BBD was employed to investigate the effects of printing speed, nozzle temperature, raster angle, and layer height on the mechanical properties. The entropy weight method assigned objective weights of 0.4924, 0.0901, and 0.4805 to the three properties, yielding a comprehensive score as the evaluation index. Unlike conventional approaches that rely solely on software-recommended optima from RSM models, this study critically compares two decision routes: secondary RSM modeling followed by continuous optimization versus direct ranking of BBD experimental data. Direct ranking identified the optimal parameter set (90 mm/s, 200 °C, 0° raster angle, 0.2 mm layer height) with a comprehensive score of 0.9614, surpassing the RSM-recommended route (70 mm/s, 200 °C, 0° raster angle, 0.2 mm layer height; score 0.7366) by 30.5%. This comparison demonstrates that, in strongly nonlinear FDM processes, software-based continuous optimization may converge to mathematically conservative suboptimal regions, whereas direct ranking of discrete experimental data preserves full physical responses and captures higher-order synergistic effects that the RSM model fails to account for. ANOVA results further confirm that raster angle is the most influential factor affecting the comprehensive score. Overall, this work bridges material formulation and printing parameter optimization for PLA/TPU blends, offering a validated strategy for high-performance FDM manufacturing.

## 1. Introduction

Additive manufacturing (AM) is revolutionizing traditional production paradigms by enabling rapid prototyping, on-demand fabrication, and geometric complexity that is unattainable through subtractive or formative processes. According to ISO/ASTM standard 52900-15 [1], AM technologies are broadly classified into seven main categories: vat photopolymerization, material jetting, binder jetting, powder bed fusion, sheet lamination, directed energy deposition, and material extrusion. Among these, fused deposition modeling (FDM)—which falls under the material extrusion category—has attracted considerable attention due to its low equipment cost, operational simplicity, material versatility, and minimal post-processing requirements, showing great potential in aerospace lightweighting, patient-specific biomedical devices, and customized high-end consumer products [2,3,4].

Polylactic acid (PLA), a bio-derived and biodegradable polyester, is among the most commonly employed FDM filament materials, owing to its favorable stiffness, printability, and compatibility with global sustainability initiatives [5]. The mechanical performance of FDM-printed PLA components is highly dependent on printing parameters such as layer height, extrusion temperature, infill density, and printing speed. For instance, Frunzaverde et al. [6] systematically investigated the effects of layer height and filament color on the dimensional accuracy and tensile strength of FDM-printed PLA specimens, revealing that smaller layer heights improve surface quality, yet this does not always translate into higher tensile strength. Similarly, Shashikumar and Sreekanth [7] examined the influence of multiple FDM parameters on the tensile properties of thermoplastic parts, demonstrating that extrusion temperature and layer thickness are among the most significant factors governing mechanical performance. Despite these processing advances, the inherent brittleness of PLA—characterized by limited elongation at break (typically <5%) and poor impact resistance—severely restricts its application in load-bearing and high-toughness scenarios. This mechanical shortcoming originates from the rigid chain architecture and slow segmental relaxation of PLA in the glassy state, which induce localized stress concentration and catastrophic brittle fracture under external loading [5,6,7].

Blending with a ductile polymer phase has been widely adopted to overcome the brittleness of PLA. Among various toughening agents, thermoplastic polyurethane (TPU)—an elastomer combining high elasticity and low crystallinity—has attracted considerable interest due to its partial miscibility with PLA and favorable interfacial compatibility [8,9]. Yurtbasi et al. [8] systematically evaluated the influence of TPU type and concentration on the rheological, mechanical, and printing-related properties of PLA/TPU blends, demonstrating that TPU content profoundly governs melt flow behavior and interlayer adhesion during FDM. Zhou et al. [9] developed multi-stimuli responsive shape memory PLA/TPU/carbon nanotube composites, highlighting the versatility of this blend system for functional applications. Rahmatabadi and co-workers conducted comprehensive investigations on FDM-printed PLA/TPU blends: they characterized the fracture toughness and mechanical properties at different component ratios [10], evaluated the shape memory effect as a function of PLA concentration and programming conditions [11], and further optimized the shape memory performance using Box–Behnken response surface methodology [12]. Ladakhan et al. [13] extended this research to PLA/TPU-based metastructures, examining their mechanical, thermal, and shape memory properties. Hamidi et al. [14] investigated the coupled effects of TPU loading and printing parameters on the thermal and mechanical performance of PLA/TPU blends, providing valuable insights into the process–structure–property relationships in FDM-printed parts. Collectively, these studies [8,9,10,11,12,13,14] establish a consensus that PLA/TPU blending effectively tailors phase morphology and crystallization behavior, thereby imparting substantially improved impact resistance and ductility relative to neat PLA.

Among various toughening strategies, TPU is selected as the blending phase for four principal reasons. First, polar interactions between the ester groups of PLA and the urethane segments of TPU impart intrinsically favorable interfacial compatibility, thereby strengthening interphase bonding without requiring additional compatibilizers [8,9,15]. Second, TPU shares an overlapping processing temperature window (190–220 °C) with PLA for standard FDM fabrication, whereas alternative toughening polymers such as polycaprolactone (PCL) and poly(butylene adipate-co-terephthalate) (PBAT) necessitate substantial adjustments to printing parameters [16]. Third, unlike nanofiller reinforcement systems—including nanoclay, carbon nanotubes, and cellulose nanofibers—TPU melt blending avoids filler agglomeration, which would otherwise deteriorate printability and mechanical performance. Fourth, the extensive literature on PLA/TPU blends [10,11,12,13,14] offers a robust foundation for cross-comparison between our experimental findings and previous studies. Furthermore, recent work by Goh et al. [17] on pellet-based printing of PLA/TPU blends has elucidated the critical role of interfacial properties in determining the mechanical performance of multi-material printed parts, reinforcing the viability of the PLA/TPU system for multifunctional applications.

Despite these advantages, the pronounced differences in molecular chain structure and polarity between rigid PLA and flexible TPU render the blend compatibility highly susceptible to compositional variations. Ferreira et al. [15] systematically investigated the effect of blend ratio on the thermal, mechanical, and shape memory properties of PLA/TPU bio-blends, revealing that phase morphology and crystallinity evolve substantially with TPU content, which in turn directly dictates the thermo-mechanical response. Such compositional variations induce marked changes in filament microstructure—including phase dispersion homogeneity, interfacial adhesion, and crystalline morphology—which critically govern the melt rheological behavior and solid-state mechanical response during FDM, thereby ultimately determining the mechanical properties of printed parts [12,13,14,15].

Despite considerable research on FDM parameter optimization and PLA/TPU blend modification [6,7,8,15], three critical research gaps persist. First, most studies have addressed FDM parameters and PLA/TPU blending ratios separately, with limited attention to their synergistic effects on mechanical performance. For instance, while Hamidi et al. [14] examined the individual effects of TPU loading and printing parameters on thermal and mechanical performance, their experimental design did not systematically explore the interactions between these two factor groups. Likewise, the comprehensive review by Ali et al. [5] on FDM of polymer blends and composites concluded that the coupled effects of blend composition and printing parameters on overall mechanical properties—particularly the balanced enhancement of tensile strength, ductility, and flexural resistance—remain insufficiently understood. This absence of integrated studies represents a critical knowledge gap, as optimal blend composition may vary with printing conditions, and vice versa [17,18].

Second, existing research predominantly focuses on macroscopic mechanical characterization, with limited efforts devoted to establishing quantitative correlations between filament microstructure and the final mechanical behavior of printed parts. Although Hamidi et al. [14] investigated the effects of TPU loading and printing parameters on tensile properties, and their subsequent work [19] characterized the shape memory performance of the same system, both studies adopted single-factor designs without considering parameter interactions or establishing quantitative linkages between microstructural evolutions—including phase uniformity, interfacial adhesion, and crystallization behavior—and the resulting mechanical response. Likewise, the roles of interlayer adhesion [20] and microscale deformation mechanisms [21] in governing tensile and flexural performance have rarely been incorporated into multi-objective optimization frameworks for PLA/TPU blends. Ruwais et al. [20] reported that PLA–TPU laminated composites exhibit limited interlayer adhesion due to intrinsic surface-energy mismatch, with interfacial optimization remaining a primary constraint for further performance gains. Rezaei et al. [21] developed a hierarchical nano-to-microscale model for 3D-printed PLA, demonstrating that microscale deformation mechanisms critically influence the overall mechanical behavior. Nevertheless, how blend ratio tailors the microstructure to govern mechanical properties remains poorly understood [15,19].

Third, most optimization efforts rely on single-objective approaches that target individual mechanical indicators—tensile strength alone, for instance—rather than addressing multiple performance criteria concurrently. Such practices inherently preclude comprehensive mechanical property optimization [22,23]. For example, Kumar et al. [22] optimized FDM printing parameters solely for tensile properties using ASTM D638 standard samples, overlooking the trade-offs between strength and ductility. As reviewed by Drégelyi-Kiss [23], although multi-objective optimization methods exist, their application to polymer blend systems for achieving balanced mechanical performance remains limited. Furthermore, while Mi and Sohi [24] and Chahdoura et al. [25] successfully applied response surface methodology to optimize FDM part quality and mechanical performance, these studies concentrated on single-material systems, leaving the multi-objective optimization of PLA/TPU blend formulations largely unexplored.

To bridge these gaps, response surface methodology (RSM) serves as an effective tool for multi-objective optimization of material formulations and process parameters. Among various RSM designs, the Box–Behnken design (BBD) offers distinct advantages, including fewer experimental runs, higher efficiency, and the capability to fit quadratic models while avoiding extreme factor combinations [26]. BBD has been widely employed in FDM process and formulation optimization, demonstrating its effectiveness in establishing predictive models and identifying optimal parameter combinations [24,25]. Building on these methodologies, this study integrates single-factor experiments with BBD within a synergistic framework to systematically investigate the coupled effects of PLA/TPU blend ratio and FDM printing parameters on the comprehensive mechanical properties of printed parts.

Complementary to the above gaps, a notable methodological issue has been overlooked in existing RSM-based studies. These studies have consistently relied on software-recommended optima from RSM models, assuming that such recommendations represent truly optimal solutions. However, whether these software-based optima remain valid in strongly nonlinear FDM processes—where parameter interactions are complex and responses are often non-monotonic—has yet to be systematically verified against direct experimental evidence. This verification is essential, as it provides critical guidance for selecting between software recommendations and experimental observations when the two diverge.

**Figure 1:**
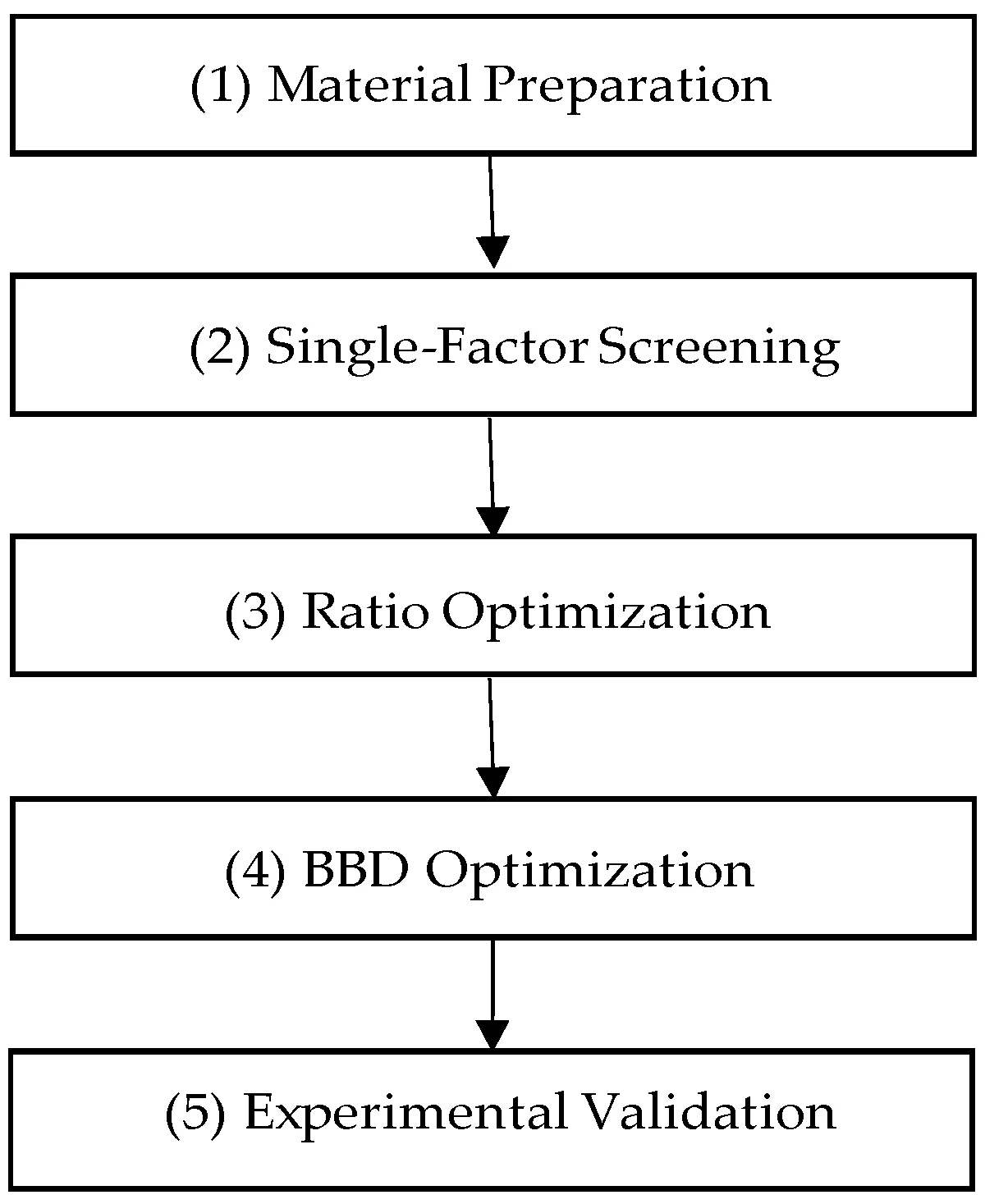
Methodological framework of this study, illustrating the five-stage sequential experimental procedure: (1) material preparation and filament fabrication; (2) single-factor experiments for blend ratio and parameter screening; (3) identification of the optimal PLA/TPU blend ratio; (4) BBD and ANOVA for multi-parameter optimization; and (5) experimental validation of the optimized parameters.

To bridge these gaps and resolve the methodological concern noted above, this study establishes a multi-objective optimization framework for FDM-printed PLA/TPU blends. The experimental strategy proceeds in two sequential steps: single-factor experiments to determine the optimal blend ratio and screen critical printing parameters, followed by a four-factor, three-level BBD to systematically investigate the effects of printing speed, nozzle temperature, raster angle, and layer height on tensile strength, elongation at break, and flexural strength. The novelty of this work lies in three aspects. First, unlike most studies that treat material formulation and printing conditions as independent variables, we introduce a hierarchical sequential strategy that bridges material design and mechanical performance modulation through a stepwise optimization logic. Second, while RSM has been widely applied in FDM optimization, the critical comparison between software-recommended optima and direct ranking of experimental data—which our results indicate as the more effective approach for the present PLA/TPU system—has not been previously reported. Third, the systematic integration of single-factor experiments, BBD, entropy-weighted multi-objective aggregation, and comparative validation provides a robust and practically applicable framework for high-performance FDM manufacturing. The overall experimental workflow is illustrated in Figure 1. The remainder of this paper is structured as follows. Section 2 describes the materials, specimen preparation, experimental design, and characterization methods. Section 3 presents the experimental results, including single-factor experiments, BBD analysis, response surface modeling, and validation. Section 4 discusses the findings, addresses the underlying mechanisms, acknowledges limitations, and outlines future directions. Section 5 concludes the paper with a summary of key findings and their implications.

## 2. Materials and Methods

### 2.1. Materials

PLA pellets (grade 2003D) were purchased from NatureWorks LLC (Plymouth, MN, USA). TPU pellets (grade 1195A) were supplied by BASF SE (Ludwigshafen am Rhein, Germany). The key physical and mechanical properties of the raw materials, as provided by the supplier technical datasheets, are summarized in Table 1.

### 2.2. Equipment and Instruments

The material preparation and specimen fabrication were carried out using an electronic balance (ML3002, Meilen, China; 3000 g capacity, 0.01 g readability), an electrical thermostatic drying oven (101-2A, Shaoxing Luyue Instrument Co., Ltd., Shaoxing, China), a micro-conical twin-screw extruder (SJSZ-10A, Wuhan Ruiming Experimental Instrument Co., Ltd., Wuhan, China), and a micro-drawing winder (FQJS-100, Wuhan Ruiming Experimental Instrument Co., Ltd., Wuhan, China). FDM printing was performed using a desktop 3D printer (Bambu Lab P2S, Shenzhen Tuozhu Technology Co., Ltd., Shenzhen, China). The crystallinity of the specimens was analyzed using an X-ray diffractometer (PANalytical Empyrean, PANalytical, Almelo, The Netherlands). Mechanical testing was performed using a universal testing machine (Shimadzu AGS-X, Shimadzu Corporation, Kyoto, Japan). FTIR spectra were recorded using a Fourier transform infrared spectrometer (Thermo Scientific Nicolet iS20, Thermo Fisher Scientific, Waltham, MA, USA). For morphological observations, the cryo-fractured surfaces were sputter-coated with gold using an ion sputtering coater (Hezao GMC-1500, Hezao, Dongguan, China) and subsequently examined using a field-emission scanning electron microscope (Zeiss Sigma300, Zeiss, Oberkochen, Germany).

### 2.3. Sample Preparation

PLA and TPU pellets were weighed using an electronic balance and mechanically blended for 50 min to ensure homogeneous dispersion. The blended mixtures were then dried in an electrical thermostatic oven at 60 °C for 8 h to remove residual moisture. Subsequently, the dried PLA/TPU blends with weight ratios of 85:15, 80:20, 75:25, and 70:30 were fed into a conical twin-screw extruder for filament fabrication. The extrusion temperatures along the barrel were set to 100, 120, 140, 175, and 180 °C. The melts were extruded and drawn at a screw speed of 20 r/min. The filament diameter was monitored periodically using a micrometer screw gauge, and the winding speed was adjusted manually to maintain the diameter within 1.65–1.75 mm. The fabricated filaments were then wound onto spools using a filament winder.

**Figure 2:**
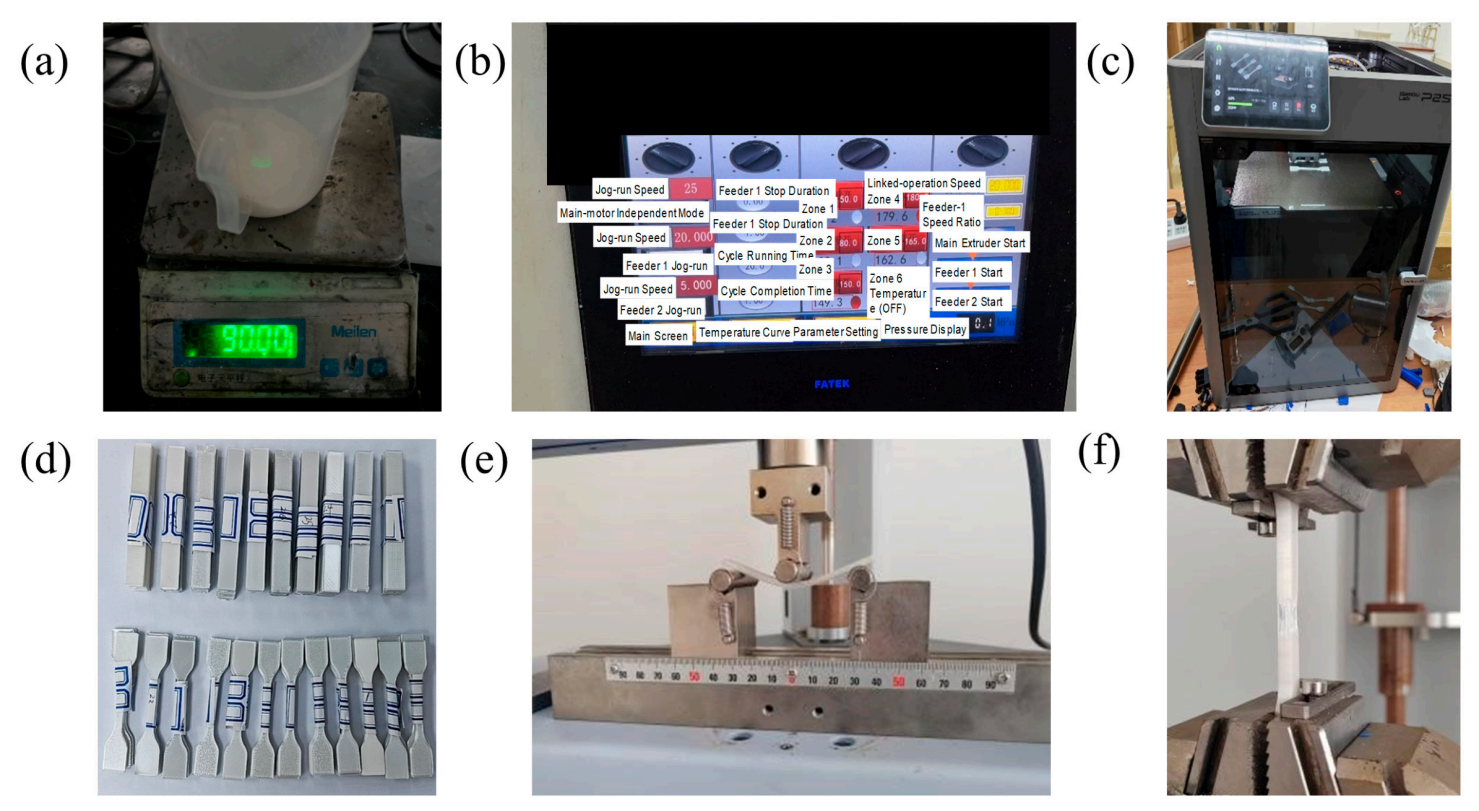
Fabrication process of specimens: (**a**) raw material weighing; (**b**) filament extrusion via twin-screw extruder; (**c**) FDM printing process; (**d**) printed specimens (three replicates per group, arranged by different processing levels); (**e**) flexural test; (**f**) tensile tests.

Tensile test specimens were prepared according to the Type 1BA dumbbell-shaped specimens specified in GB/T 1040.2-2022 [27], with a gauge length of 25 mm, gauge width of 5 mm, thickness of 4 mm, and overall length of 75 mm. Flexural test specimens were prepared according to the rectangular bar specimens specified in GB/T 9341-2008 [28], with key dimensions of length 80 mm, width 10 mm, and thickness 4 mm. All specimens were printed using the as-prepared filaments via the FDM process. After printing, the specimens were naturally cooled within the enclosed chamber for approximately 40 min before being removed. Subsequently, all specimens were conditioned at ambient temperature for 24 h to eliminate residual thermal effects and ensure complete relaxation, after which mechanical testing was performed. Three replicates were printed for each experimental condition. The overall experimental procedure is illustrated in Figure 2.

### 2.4. Performance Testing and Structural Characterization

#### 2.4.1. Mechanical Property Testing

Tensile testing was conducted on an electronic universal testing machine in accordance with GB/T 1040.2-2022 at a crosshead speed of 20 mm/min, which falls within the standard-recommended speed range for plastics. Tensile strength (MPa) and elongation at break (%) were recorded. Flexural testing was performed on the same testing machine at a crosshead speed of 2 mm/min according to GB/T 9341-2008, and flexural strength (MPa) was recorded. Three specimens were tested for each group, and the results were reported as arithmetic means.

#### 2.4.2. Fourier Transform Infrared (FTIR) Spectroscopy

Fourier transform infrared (FTIR) spectra were recorded using a Thermo Scientific Nicolet iS20 spectrometer (Waltham, MA, USA) equipped with an attenuated total reflectance (ATR) accessory. The spectra were collected over a wavenumber range of 400–4000 cm^−1^ with 32 scans per sample to investigate the changes in characteristic absorption peaks of the blend system.

#### 2.4.3. X-Ray Diffraction (XRD) Analysis

X-ray diffraction (XRD) analysis was performed on the specimens using an X-ray diffractometer to investigate the crystalline structure. The measurements were conducted under the following conditions: Cu Kα radiation (λ = 0.15418 nm), generator voltage of 40 kV, current of 40 mA, scanning range of 2θ = 5–90°, scanning speed of 10°/min, and cumulative scanning count of 16. The crystallinity ($X_{c}$) of the specimens was calculated using Equation (1),

(1) $$X_{c}=\frac{A_{c}}{A_{C}+KA_{a}}\times 100\%$$

where $A_{c}$ and $A_{a}$ are the integrated intensities of the crystalline diffraction peaks and the amorphous halo, respectively, over the integration range, and *K* is the experimental calibration factor.

#### 2.4.4. Scanning Electron Microscopy (SEM) Observation

The morphology of the cryo-fractured surfaces of the specimens was observed using a scanning electron microscope. Prior to observation, the cryo-fractured samples were mounted onto conductive sample holders and sputter-coated with gold using an ion sputter coater (sputtering time: 30 s, current: 10 mA) to eliminate charge accumulation on the surface of the non-conductive materials. The micrographs were acquired using a secondary electron detector at an acceleration voltage of 5 kV.

### 2.5. Experimental Design

This study adopted a combined strategy of single-factor experiments and Box–Behnken design (BBD) response surface methodology to systematically investigate the effects of processing parameters and blend ratio on the mechanical properties of FDM-printed parts. Tensile strength (MPa), elongation at break (%), and flexural strength (MPa) were selected as the core evaluation indicators to achieve synergistic optimization of material formulation, processing parameters, and mechanical properties. All experiments were performed in triplicate for each condition, and the results were reported as mean ± standard deviation (*SD*).

#### 2.5.1. Single-Factor Preliminary Experiments

Following the principle of control variables, single-factor experiments were conducted to examine the individual effects of printing speed (50–90 mm/s), nozzle temperature (190–210 °C), raster angle (0–90°, the orientation of the deposited filament within each layer), layer height (0.1–0.3 mm), and PLA/TPU blend ratio (85:15, 80:20, 75:25, and 70:30 by weight) on the mechanical properties of the printed parts. All specimens were printed using a nozzle diameter of 0.4 mm and an extrusion multiplier of 1.0, with the printing chamber maintained at 25 ± 2 °C.

The selection criteria for each processing parameter were as follows. Printing speed (*A*) was set at 50, 70, and 90 mm/s based on the rheological characteristics of the PLA/TPU blend melt; excessive speed leads to insufficient melt spreading and interlayer defects, while excessively low speed may cause thermal degradation and significantly increases printing time. Nozzle temperature (*B*) was set at 190, 200, and 210 °C according to the optimal melting window of the blend; the default 220 °C for neat PLA was excluded, as temperatures above 210 °C induce thermal oxidation of TPU soft segments and PLA chain scission, leading to significant mechanical deterioration [13,15]. Raster angle (*C*)—defined as the angle between the internal filling paths and the specimen longitudinal direction—was investigated at 0°, 45°, and 90° to cover different stress transfer pathways. Layer height (*D*) was set at 0.1, 0.2, and 0.3 mm considering the “staircase effect” and interlayer contact area, balancing printing efficiency and structural densification.

Through these single-factor experiments, the preliminary optimal ranges of FDM processing parameters for each blend ratio were determined, and a promising range of PLA/TPU blend ratios was screened out, providing a basis for the subsequent response surface optimization.

#### 2.5.2. Response Surface Optimization Experiments

Based on the single-factor results, a four-factor, three-level Box–Behnken design (BBD) was employed to systematically investigate the combined effects of printing speed (*A*), nozzle temperature (*B*), raster angle (*C*), and layer height (*D*) on the mechanical properties of PLA/TPU blends under the optimal blend ratio. Combined with analysis of variance (ANOVA) at a significance level of α = 0.05, this stage quantitatively evaluated the significance of individual process parameters and their interactions. Quadratic regression models were constructed for tensile strength, elongation at break, and flexural strength to predict and optimize the printing parameters. The reliability of the models was confirmed through validation experiments conducted under the predicted optimal conditions. This stage was intended to compensate for the inherent limitation of single-factor experiments in assessing multi-parameter synergistic effects, while also revealing the coupling mechanism between blend composition and printing conditions.

## 3. Results

### 3.1. Design and Implementation of Single-Factor Experiments

#### 3.1.1. Design of the Single-Factor Experimental Scheme

Single-factor experiments were conducted to systematically investigate the independent effects of PLA/TPU blend ratios (85:15, 80:20, 75:25, and 70:30 by weight) and key printing parameters—printing speed (*A*), nozzle temperature (*B*), raster angle (*C*), and layer height (*D*)—on the mechanical properties of FDM-printed parts. The experiments strictly followed the principle of control variables, wherein only one factor was varied at a time while all other printing parameters and the blend ratio were kept constant, allowing for the precise quantification of the individual effects of each factor on tensile strength (MPa), elongation at break (%), and flexural strength (MPa). Based on the results obtained from the single-factor experiments, bar charts (presented as mean ± *SD*) were constructed to illustrate the influence of each factor on the three mechanical properties. The resulting trends were analyzed to determine the significance and priority order of each factor’s effect on the mechanical performance, thereby providing a rational basis for selecting factor levels in the subsequent response surface optimization experiments.

Table 2 summarizes the level design of the four processing parameters in the single-factor experiments, with selection criteria as follows: printing speed was set at 50, 70, and 90 mm/s based on the rheological characteristics of the PLA/TPU blend melt; nozzle temperature was set at 190, 200, and 210 °C according to the optimal melting window (190–210 °C) of the blend, while the default 220 °C was excluded [15,16]; raster angles of 0°, 45°, and 90° were selected as three typical orientations; and layer height was set at 0.1, 0.2, and 0.3 mm considering the material properties and equipment precision [29].

#### 3.1.2. Single-Factor Experimental Results and Analysis

**Figure 3:**
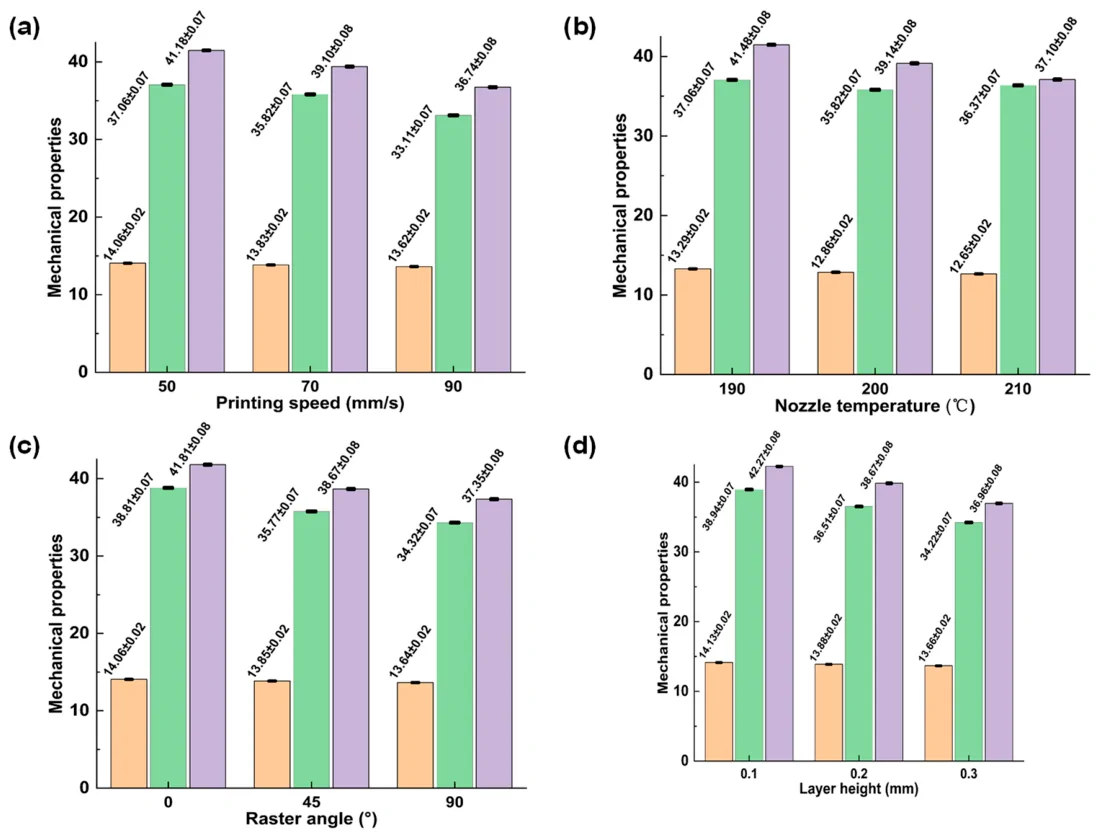
Effects of FDM process parameters on the mechanical properties of PLA/TPU (80:20) blends: (**a**) printing speed, (**b**) nozzle temperature, (**c**) raster angle, and (**d**) layer height. Data are presented as mean ± *SD* (*n* = 3), with mean values shown above the bars. Orange, green, and purple bars represent elongation at break (%), tensile strength (MPa), and flexural strength (MPa), respectively.

**Figure 4:**
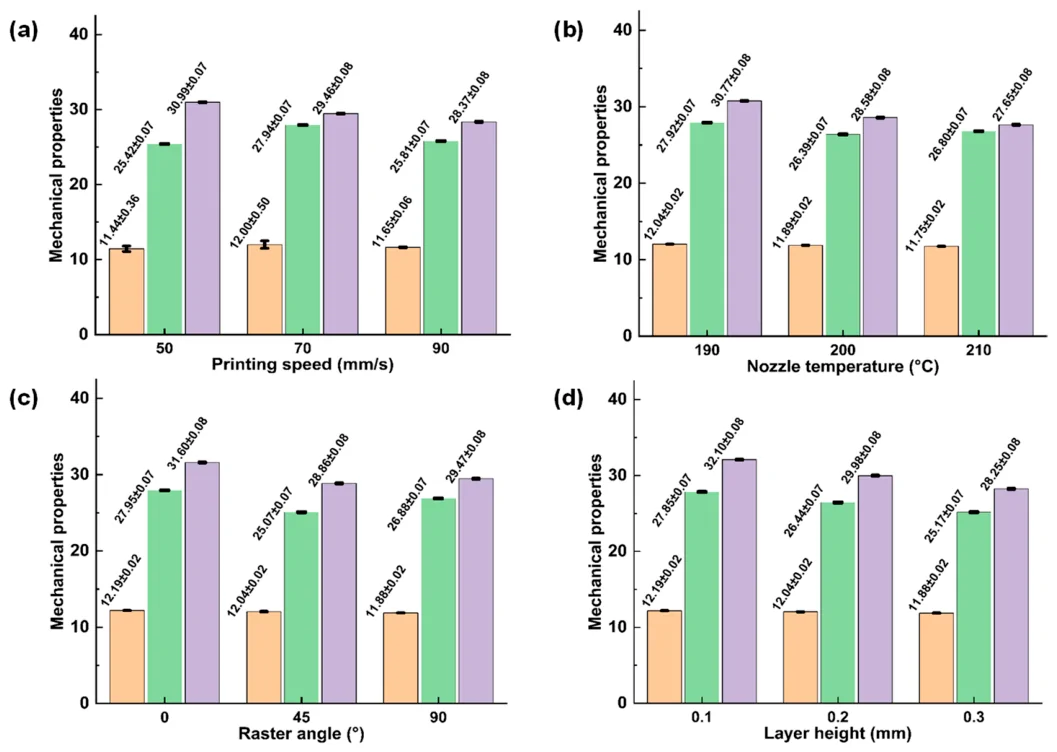
PLA/TPU (70:30) blends: (**a**) printing speed, (**b**) nozzle temperature, (**c**) raster angle, and (**d**) layer height. Data presentation and testing standards are identical to those in Figure 3.

**Figure 5:**
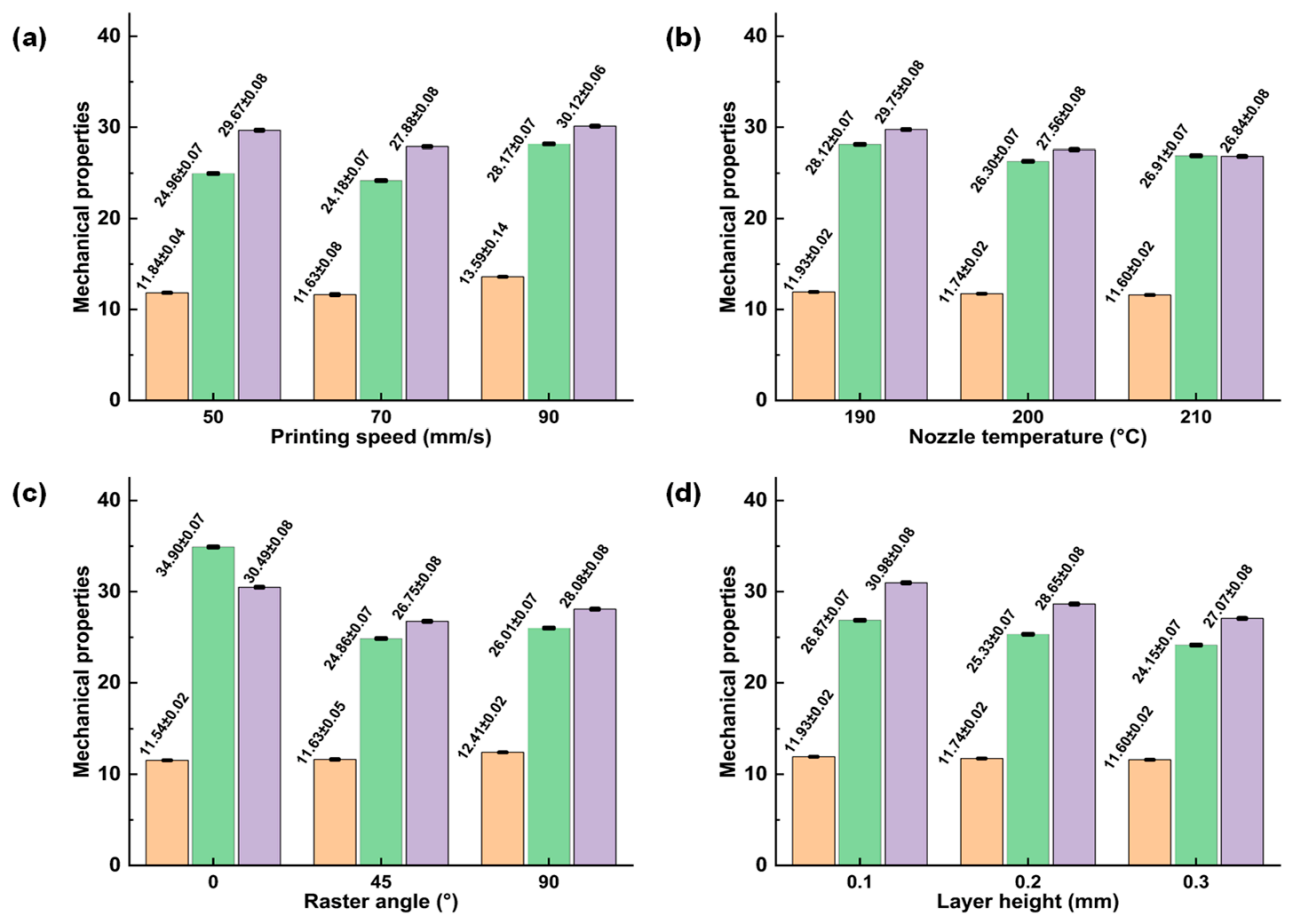
PLA/TPU (75:25) blends: (**a**) printing speed, (**b**) nozzle temperature, (**c**) raster angle, and (**d**) layer height. Data presentation and testing standards are identical to those in Figure 3.

**Figure 6:**
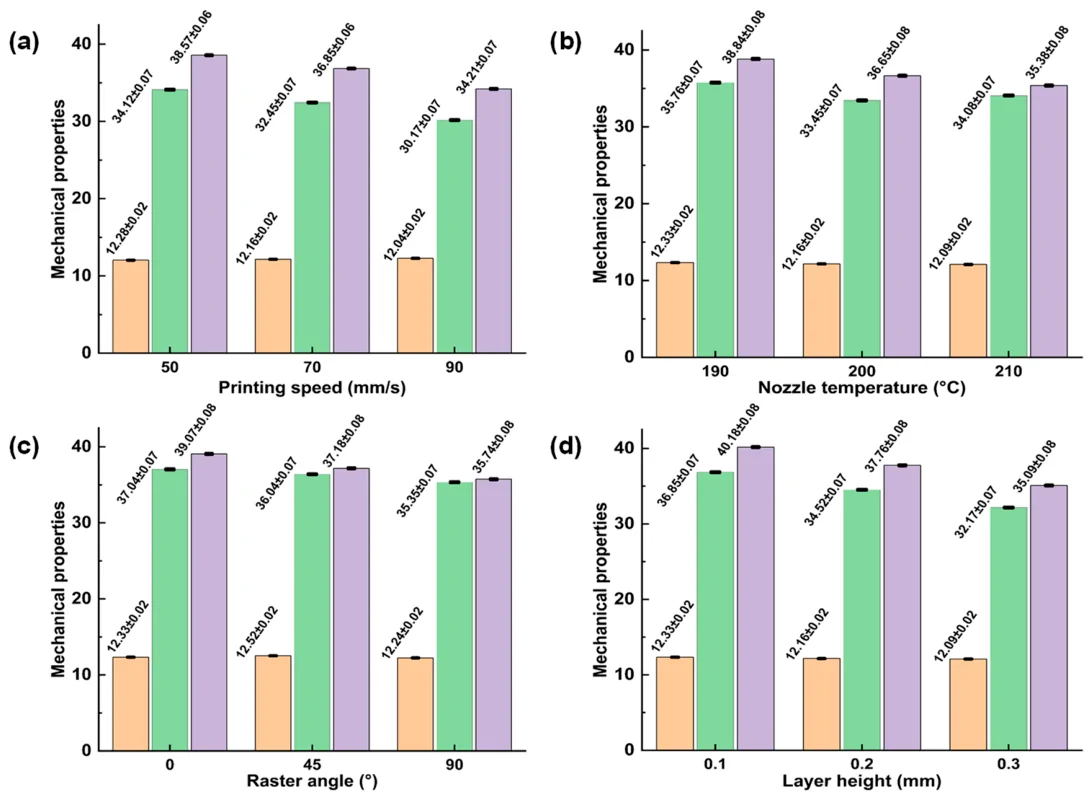
PLA/TPU (85:15) blends: (**a**) printing speed, (**b**) nozzle temperature, (**c**) raster angle, and (**d**) layer height. Data presentation and testing standards are identical to those in Figure 3.

This section presents the single-factor experimental results for the PLA/TPU (80:20) blend system as a representative formulation. Figure 3a–d illustrate the effects of printing speed, nozzle temperature, raster angle, and layer height on the tensile strength (MPa), elongation at break (%), and flexural strength (MPa). Data are presented as mean ± *SD*. The corresponding results for the other blend ratios (85:15, 75:25, and 70:30) are summarized in Figure 3, Figure 4, Figure 5 and Figure 6 and discussed briefly.

Printing speed (Figure 3a): In the range of 50–90 mm/s, all three mechanical properties decreased continuously with increasing printing speed. The optimal comprehensive performance was observed at 50 mm/s. Increasing speed was accompanied by reductions in all three properties, which may be associated with insufficient fusion time and the formation of interlayer defects [30,31].

Nozzle temperature (Figure 3b): The mechanical properties exhibited a trend of first increasing and then decreasing with rising temperature, with tensile and flexural strengths reaching their optimum at 200 °C. At 190 °C, mechanical properties were inferior to those at 200 °C [30]. At 210 °C, all three mechanical properties decreased, with elongation at break showing a sustained decline [29].

Raster angle (Figure 3c): The 0° infill specimens exhibited superior tensile strength, flexural strength, and elongation at break compared to 45° and 90° specimens [32,33]. At 45°, the properties were intermediate between those at 0° and 90° [34]. At 90°, the lowest mechanical properties were observed [32]. Overall, 0° was identified as the optimal raster angle among the three orientations tested.

Layer height (Figure 3d): As the layer height increased from 0.1 mm to 0.3 mm, all three mechanical properties decreased simultaneously. The highest mechanical properties were observed at 0.1 mm [35]. Increasing layer height resulted in lower mechanical properties [31,36].

In summary, the optimal parameter combination determined by the single-factor experiments for the PLA/TPU (80:20) blend system was: printing speed of 50 mm/s, nozzle temperature of 200 °C, raster angle of 0°, and layer height of 0.1 mm. Across all tested parameters, the trends of tensile strength and flexural strength were closely aligned with each other and generally consistent with that of elongation at break.

As shown in Figure 4, the regulatory patterns of the four processing parameters on the mechanical properties at the 70:30 blend ratio were generally consistent with those at the 80:20 ratios, though differences were observed in the optimal parameter values. Specifically, tensile strength and flexural strength first increased and then decreased with increasing printing speed, reaching their peaks at 70 mm/s (whereas a continuous decline was observed for the 80:20 ratio). The optimal nozzle temperature remained at 200 °C for both ratios, and the optimal raster angle and layer height were consistently 0° and 0.1 mm, respectively. In summary, the optimal parameter combination for the 70:30 blend ratio was determined as: printing speed of 70 mm/s, nozzle temperature of 200 °C, raster angle of 0°, and layer height of 0.1 mm.

As shown in Figure 5, the optimal levels of raster angle and layer height at the 75:25 blend ratio remained consistent with those of the aforementioned ratios (0° and 0.1 mm, respectively). The main differences were observed in the other two parameters: tensile strength exhibited a monotonic increasing trend with printing speed, reaching its optimum at 90 mm/s, which differs from the monotonic decrease at 80:20 and the initial increase followed by a decrease at 70:30; the optimal nozzle temperature decreased to 190 °C, with the mechanical properties continuously deteriorating as the temperature increased. Consequently, the optimal parameter combination for the 75:25 blend ratio was determined as: printing speed of 90 mm/s, nozzle temperature of 190 °C, raster angle of 0°, and layer height of 0.1 mm.

As shown in Figure 6, the optimal levels of raster angle and layer height at the 85:15 blend ratio remained 0° and 0.1 mm, consistent with the aforementioned ratios. The printing speed exhibited a monotonic decreasing trend with an optimal value of 50 mm/s, following the same pattern as the 80:20 ratio. The optimal nozzle temperature was 190 °C, with mechanical properties continuously deteriorating as the temperature increased, which aligned with the behavior observed for the 75:25 ratio. In summary, the optimal parameter combination for the 85:15 blend ratio was determined as: printing speed of 50 mm/s, nozzle temperature of 190 °C, raster angle of 0°, and layer height of 0.1 mm.

Based on the single-factor experimental results described above, the mechanical properties and corresponding optimal parameter combinations for the four PLA/TPU blend ratios are summarized in Table 3. The results reveal that the optimal levels of raster angle and layer height were highly consistent across all blend ratios (0° and 0.1 mm, respectively), whereas the optimal printing speed and nozzle temperature varied noticeably with the blend ratio. Considering the overall mechanical performance, the 80:20 blend achieved the best comprehensive properties at 50 mm/s and 200 °C, and was therefore selected as the formulation for subsequent response surface optimization experiments.

### 3.2. Microstructural Characterization and Mechanistic Verification

#### 3.2.1. FTIR Analysis

**Figure 7:**
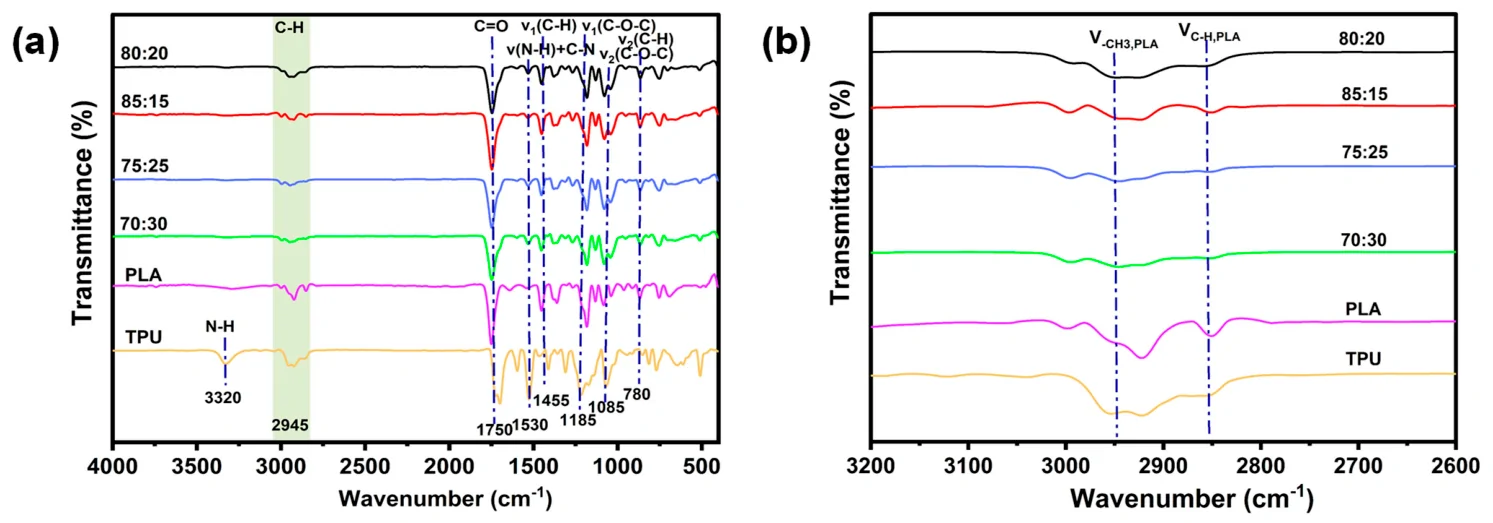
(**a**) Full-range FTIR transmittance spectra (4000–400 cm^−1^) of neat PLA, neat TPU, and PLA/TPU blends with various mass ratios. The principal absorption bands of PLA are observed at 1750 cm^−1^ (C=O stretching), 2995 and 2945 cm^−1^ (C–H stretching), and 1185 and 1085 cm^−1^ (C–O–C stretching). Characteristic peaks of TPU are located at 3320 cm^−1^ (N–H stretching), 1725 cm^−1^ (C=O stretching), and 1530 cm^−1^ (amide II band). (**b**) Magnified FTIR spectra collected over the wavenumber range of 2600–3200 cm^−1^. The dashed lines denote the characteristic $v_{–CH^{3}}$ and $v_{C–H}$ stretching vibration peaks of PLA chains.

Fourier transform infrared (FTIR) spectroscopy was employed to investigate the molecular interactions and interfacial compatibility between PLA and TPU. Figure 7 shows the FTIR spectra of neat PLA, neat TPU, and their blends over the range of 4000–400 cm^−1^ in transmittance mode.

The PLA spectrum exhibits characteristic peaks of linear polyesters: a sharp C=O stretching vibration at 1750 cm^−1^ (ester carbonyl), C–H stretching bands of methyl groups at 2995 and 2945 cm^−1^, and C–O–C stretching vibrations at 1185 and 1085 cm^−1^ [37]. For neat TPU, the spectrum shows an N–H stretching band at 3320 cm^−1^ (hydrogen-bonded urethane groups), a C=O stretching peak at 1725 cm^−1^ (urethane carbonyls), an amide II band at 1530 cm^−1^ (N–H bending coupled with C–N stretching), and a C–O–C ether stretching band near 1100 cm^−1^ [38].

For all PLA/TPU blends, the FTIR spectra display a simple superposition of the characteristic peaks of both components without any new absorption bands, confirming that no chemical reaction occurs during melt blending; the two phases are physically combined and their intrinsic chemical structures remain intact [39]. As the TPU proportion increases, PLA peak intensities (1750 cm^−1^ and 1185 cm^−1^) gradually decrease while TPU peaks (3320 cm^−1^ and 1530 cm^−1^) increase, showing a positive correlation with the respective mass fractions and indicating uniform dispersion of both phases.

Notably, the 80:20 blend exhibits the most pronounced red shift of the PLA ester carbonyl peak (1750 cm^−1^, ~4–6 cm^−1^ toward lower wavenumbers) and the most significant broadening of the TPU N–H stretching band (3320 cm^−1^), while the 70:30 blend shows the weakest shifts. This indicates that the 80:20 composition achieves the strongest intermolecular hydrogen bonding between PLA ester carbonyl groups and TPU N–H groups, resulting in the most intimate interfacial adhesion. Such strong interfacial bonding improves compatibility, facilitates efficient stress transfer, and mitigates stress concentration, which are associated with the superior mechanical strength and toughness of the 80:20 blend, consistent with the mechanical performance results [39].

#### 3.2.2. X-Ray Diffraction Analysis

**Figure 8:**
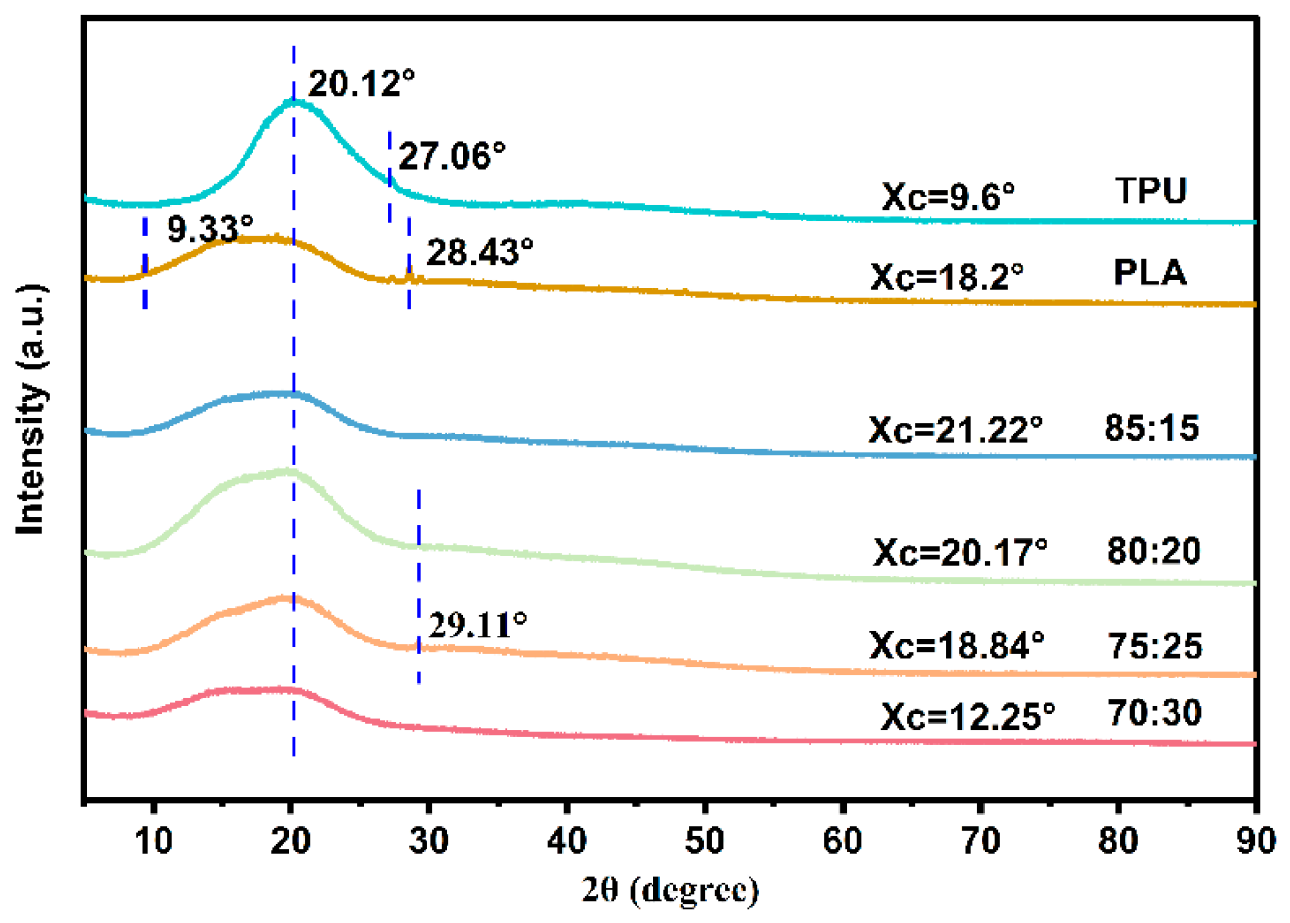
XRD patterns of neat PLA, neat TPU, and PLA/TPU blend filaments at different weight ratios (85:15, 80:20, 75:25, and 70:30). The inset table summarizes the crystallinity values derived from the integrated peak areas.

Figure 8 displays the XRD patterns of neat PLA, neat TPU, and their blends. Neat PLA exhibits sharp crystalline diffraction peaks at 16.72° and 19.08°, characteristic of its α-crystalline structure. Neat TPU shows a broad diffuse diffraction halo centered at 20.04° with a weak shoulder near 27.06°, arising from microphase separation between hard and soft segments, consistent with its crystallinity of 9.6% [37].

No new diffraction peaks emerge for any blend, confirming that PLA and TPU are physically combined without chemical reactions or co-crystallization at the interface. The primary PLA diffraction peak shifts slightly toward higher 2θ values with increasing TPU content, suggesting reduced interchain spacing due to intercalation of TPU chains that disrupt ordered PLA chain packing.

The crystallinity exhibits a non-monotonic variation with TPU loading. At 85:15, crystallinity increases from 18.2% (neat PLA) to 21.2%, indicating that appropriately dispersed TPU domains act as heterogeneous nucleation sites [38]. Further TPU addition (80:20 and 75:25) progressively reduces crystallinity to 20.2% and 18.8%, respectively, due to steric hindrance from interpenetrating TPU chains that restrict PLA segmental mobility. At 70:30, crystallinity drops sharply to 12.3%, accompanying a phase inversion where TPU becomes the continuous matrix, confirming that excessive TPU severely suppresses PLA crystallization [39].

The XRD results structurally validate the superior performance of the 80:20 blend. First, its crystallinity of 20.2%—marginally lower than 85:15 (21.2%) but distinctly higher than 75:25 (18.8%) and 70:30 (12.3%)—combined with well-defined PLA diffraction peaks, confirms that PLA remains the continuous matrix, providing a rigid load-bearing skeleton. Second, the higher TPU content in 80:20 versus 85:15 provides abundant elastomeric domains for crazing and shear yielding, delivering toughening absent in the 85:15 blend. Third, the lower TPU fraction in 80:20 compared to 75:25 reduces chain entanglements and steric hindrance, preserving PLA structural integrity during toughening modification. Overall, the 80:20 ratio achieves an optimal synergy between the rigid crystalline PLA skeleton and flexible TPU toughening phase, realizing an excellent strength–toughness balance.

#### 3.2.3. Morphological Analysis of Typical Blend Ratios

**Figure 9:**
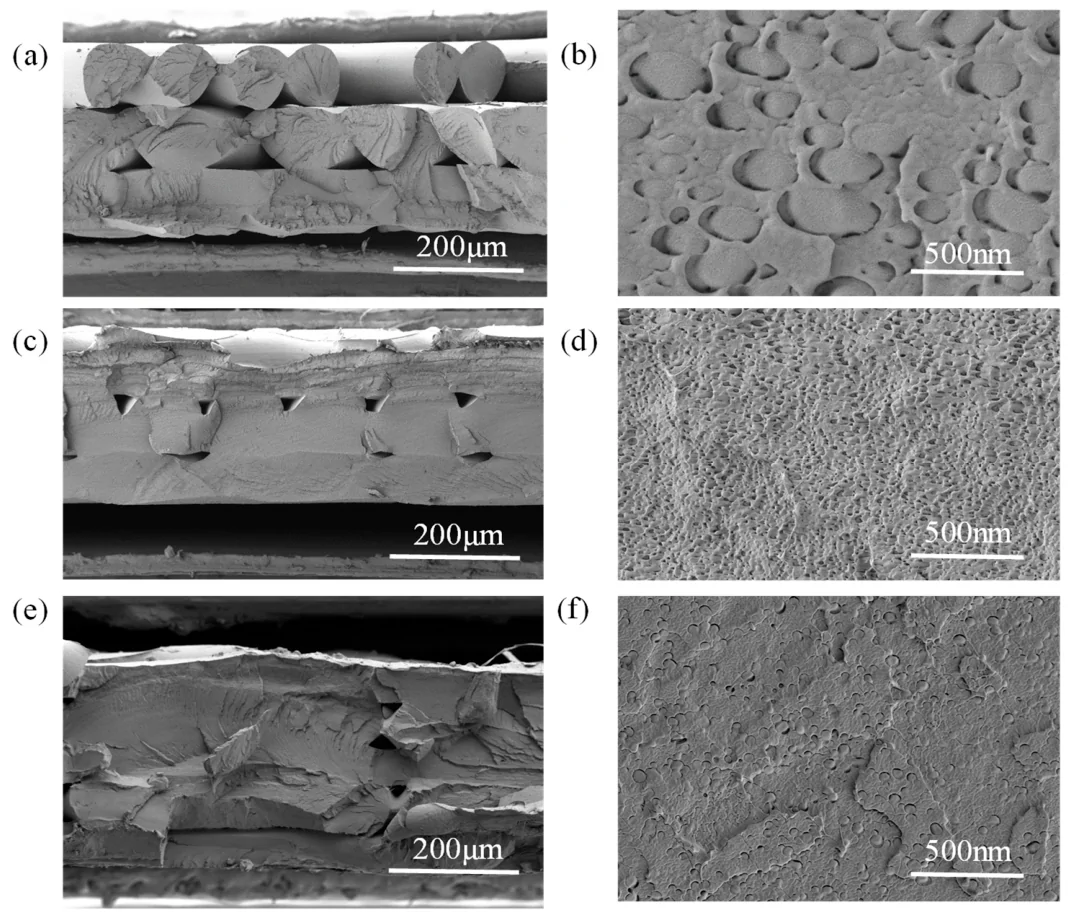
SEM images of tensile fracture surfaces: (**a**,**b**) neat PLA; (**c**,**d**) PLA/TPU = 70:30; (**e**,**f**) PLA/TPU = 80:20. (**a**,**c**,**e**) Low-magnification fracture surfaces (scale bar: 200 μm); (**b**,**d**,**f**) high-magnification morphologies of TPU dispersed phases (scale bar: 500 nm).

The mechanical testing results in Section 3.1.2 identified the 80:20 blend as the optimum and the 70:30 as the poorest performer. To elucidate the microstructural origin of this difference, three representative compositions—neat PLA (baseline), 80:20, and 70:30—were selected for SEM observation (Figure 9).

All specimens were printed under identical processing parameters (50 mm/s, 190 °C, 0° raster angle, 0.1 mm layer height) to ensure that morphological differences are solely attributable to the blend ratio, not process variations. This unified parameter set has a clear experimental basis: while the optimal printing speed varies across ratios (85:15 and 80:20 at 50 mm/s; 75:25 at 90 mm/s; 70:30 at 70 mm/s), the nozzle temperature (190 °C), raster angle (0°), and layer height (0.1 mm) remain consistent. The speed was uniformly set to 50 mm/s—the optimal speed for the majority of ratios—which also exhibits good printability for all compositions, enabling valid morphological comparison across the selected blends.

As shown in Figure 9, the fracture surfaces of the three blends exhibit distinct morphological features at both the microscale (Figure 9a,c,e) and nanoscale (Figure 9b,d,f). Neat PLA (Figure 9a,b) displays a relatively flat and smooth fracture surface with clearly distinguishable interlayer boundaries, characteristic of brittle failure with rapid crack propagation and no effective energy dissipation [14].

The 70:30 blend (Figure 9c,d) shows a substantial increase in interlayer voids and micro-defects. The high TPU content promotes agglomeration of elastomeric particles, which act as stress concentration sites that nucleate microvoids during tensile loading. At the nanoscale, a high density of closely packed TPU particles is observed, with neighboring domains coalescing into larger agglomerates, indicating severe phase separation that impairs mechanical performance [14].

In contrast, the 80:20 blend (Figure 9e,f) exhibits extensive plastic deformation with wrinkled, tear-like ridges and pronounced surface undulations—signature features of ductile failure. The PLA matrix underwent substantial plastic yielding during tensile loading, leading to higher energy dissipation prior to fracture [10]. At the nanoscale, fine, spherical TPU domains are uniformly and independently dispersed throughout the PLA matrix without significant agglomeration. These well-dispersed elastomeric particles effectively trigger shear yielding and crazing, providing a toughening effect [40].

The SEM observations thus provide direct microstructural evidence that the 80:20 blend achieves an optimum balance between rigid PLA skeletal integrity and flexible TPU toughening efficiency, supporting its enhanced tensile strength, flexural strength, and fracture toughness [14].

### 3.3. Optimization Results and Validation

#### 3.3.1. BBD Experimental Design and Results

In the preceding single-factor experiments, the effects of printing speed (*A*), nozzle temperature (*B*), raster angle (*C*), and layer height (*D*) on the mechanical properties of FDM-printed PLA/TPU composites were systematically investigated, and the blend ratio of 80:20 (PLA/TPU) was identified as the optimal formulation. To further optimize the printing parameters for this ratio, a Box–Behnken design (BBD) was employed for multi-factor process optimization based on the single-factor results. Each factor was varied at three levels, coded −1, 0, and +1, as detailed in Table 4.

The responses evaluated were tensile strength ($\sigma_{t}$), elongation at break ($\varepsilon_{b}$), and flexural strength ($\sigma_{f}$). Specimen preparation and mechanical testing were conducted according to the 29 runs of the Box–Behnken design (including 5 center-point replicates). For each run, three parallel specimens were tested, and the results are presented as the mean ± *SD*. The detailed experimental layout and the corresponding mechanical properties are provided in Table 5.

#### 3.3.2. Comprehensive Score Calculation Based on the Entropy Weight Method

As shown in Table 5, the three mechanical properties have different dimensions and exhibit markedly distinct variations across printing parameters. To objectively evaluate the overall performance of each experimental scheme, these indices must be integrated into a single comprehensive score. Since subjective weighting methods (e.g., analytic hierarchy process) rely on expert experience and may introduce bias, the entropy weight method was adopted for objective weighting.

The entropy weight method assigns higher weights to indices with greater variability, as they contain more information, and lower weights to those with limited differentiation capability. Based on the 29 experimental runs in Table 5, the weights for tensile strength, elongation at break, and flexural strength were determined as 0.4924, 0.0901, and 0.4805, respectively. This distribution reflects the actual data dispersion: tensile strength (28.81–48.14 MPa, range 19.33 MPa) and flexural strength (29.86–51.76 MPa, range 21.90 MPa) exhibit much wider ranges than elongation at break (9.70–10.85%, range 1.15%). Accordingly, tensile and flexural strengths together account for 97.29% of the total weight, while elongation at break receives only 9.01%, consistent with its limited variation.

The comprehensive score Y for each run was then calculated using Equation (2), with results listed in the last column of Table 5:

(2) $$Y_{i}=0.4294Z_{i1}+0.0901Z_{i2}+0.4805Z_{i3}$$

where $Z_{i1}$, $Z_{i2}$, and $Z_{i3}$ are the min–max normalized values of tensile strength, elongation at break, and flexural strength for the i-th run, respectively. A higher *Y* indicates superior overall mechanical performance. Run 18 exhibits the highest comprehensive score (0.9614), with both tensile strength (48.14 MPa) and flexural strength (51.56 MPa) reaching their maxima among all runs, preliminarily suggesting outstanding overall mechanical performance.

#### 3.3.3. Development and Reliability Analysis of Regression Models

A total of 29 experimental runs (including 5 replicates at the center point) were conducted in Table 5, and response surface analysis was performed using Design-Expert 12 software. The resulting quadratic polynomial regression equations for the three mechanical properties and the comprehensive score, expressed in terms of coded factors, are given in Equations (3)–(6).

(3) $$Y_{1}=35.25+1.34A-1.39C-3.96AC+4.86C^{2}$$

(4) $$Y_{2}=10.41+0.2733A-0.0558C-0.0342D+0.2925AD-0.2528C^{2}$$

(5) $$Y_{3}=39.89-1.37B-2.35C-3.54B^{2}+9.07C^{2}$$

(6) $$Y_{4}=0.4435-0.0860C+0.2767C^{2}$$

**Figure 10:**
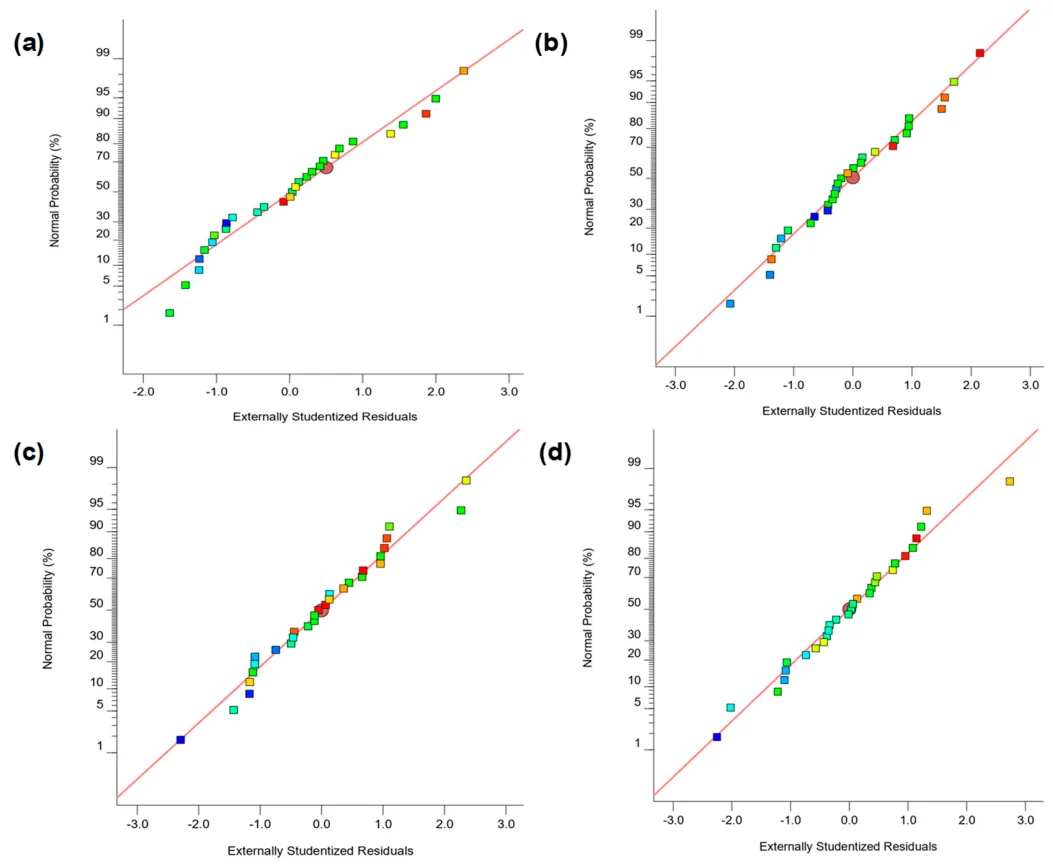
Normal probability plots of studentized residuals for: (**a**) tensile strength, (**b**) elongation at break, (**c**) flexural strength, and (**d**) comprehensive score. The colored squares and circles denote the data points for the four response variables.

To verify the reliability of the above four regression equations, residual normality analysis was performed, and the results are shown in Figure 10.

In Figure 10, the residual data points closely follow the reference line, indicating that the residuals are normally distributed. This confirms that the four polynomial regression equations are statistically valid and can be used for subsequent parameter optimization analysis.

To examine the significance of the quadratic polynomial regression models, ANOVA and significance testing were performed. Table 6, Table 7, Table 8 and Table 9 present the ANOVA results for tensile strength, elongation at break, flexural strength, and comprehensive score, respectively. To more clearly illustrate the combination of significant terms, non-significant sources of variation were removed from each table while preserving the standard hierarchical structure.

As shown in Table 6, Table 7, Table 8 and Table 9, the quadratic regression models for all response variables (tensile strength, elongation at break, flexural strength, and comprehensive score) are highly significant (model *p* < 0.01), demonstrating that the response surface methodology effectively characterizes the nonlinear relationships between printing parameters and mechanical properties. The lack-of-fit *p*-values for all models exceed 0.05, confirming no significant lack of fit and validating the adequacy of the selected models.

From the composition of significant terms in each model, distinct governing mechanisms emerge. For tensile strength, the response is primarily driven by the *AC* interaction term and *C*^2^, while the main effects of speed *A* and angle *C* are not significant (*p* > 0.1), indicating dominance of nonlinear effects. The effect size analysis further confirms that *C*^2^ exhibits a large Partial *η*^2^ of 0.436, while *AC* also shows a large effect size of 0.226, underscoring the practical significance of both the quadratic angle effect and its interaction with speed (Partial *η*^2^ values are interpreted according to Cohen‘s (1988) criteria: 0.01–0.06 indicates a small effect, 0.06–0.14 indicates a medium effect, and ≥0.14 indicates a large effect.) [41]. In contrast, the main effects of *A* and *C* yield medium-to-small effect sizes of 0.091 and 0.097, consistent with their non-significant *p*-values.

For elongation at break, the significant influences of *A*, the *AD* interaction, and *C*^2^ suggest a synergistic effect between speed and layer height alongside a significant nonlinear effect of raster angle. The Partial *η*^2^ values substantiate this interpretation: *A* exhibits the largest effect size (0.443), followed by *C*^2^ (0.285) and *AD* (0.231), all exceeding the large-effect threshold of 0.14. The main effects of *C* and *D*, however, show only small effect sizes (0.034 and 0.012, respectively), indicating negligible independent contributions.

For flexural strength, the model is mainly governed by *B*^2^ and *C*^2^, with the main effects of temperature B and angle *C* failing to reach statistical significance (*p* > 0.05). The Partial *η*^2^ analysis reveals that *C*^2^ dominates the response with a large effect size of 0.583, while *B*^2^ also shows a large effect size of 0.176. The main effect of *C* exhibits a Partial *η*^2^ of 0.140—approaching the large-effect threshold—suggesting that its engineering significance should not be overlooked despite its *p*-value of 0.059.

For the comprehensive score, the model retains only *C*^2^ (*p* < 0.001) as a significant term, while the other three main effects are excluded. The effect size analysis confirms that *C*^2^ exhibits a large Partial *η*^2^ of 0.482, whereas the main effect of *C* shows a value of 0.134, approaching the large-effect threshold. This simplified model structure indicates that raster angle *C* is the sole dominant factor in the comprehensive score response space.

This statistical result does not contradict the outstanding experimental performance of Run 18 (90/200/0/0.2) in Table 5. At the same raster angle of 0°, Runs 5 (70/200/0/0.1), 7 (70/200/0/0.3), and 18 (90/200/0/0.2) achieved comprehensive scores of 0.7270, 0.9367, and 0.9614, respectively, showing considerable variation despite identical angles. However, ANOVA tests the global average effects across the entire design space, whereas the nonlinear synergistic gain between speed and layer height at Run 18 occurs only locally and does not sufficiently alter the global response surface curvature to reach statistical significance. This phenomenon reveals the inherent limitation of low-order response surface models—statistically non-significant does not imply engineering irrelevance [42]. Response surface methodology may converge to local optima rather than the global optimum when the response surface is highly multimodal [43]. The performance peak represented by Run 18 was successfully captured through direct ranking of BBD measured data rather than through RSM-based continuous optimization.

Regarding model fitting quality, the differences between the adjusted R^2^ ($R_{\mathrm{Adj}}^{2}$) and predicted R^2^ ($R_{\mathrm{Pred}}^{2}$) for all models are less than 0.2, ruling out the risk of overfitting and demonstrating good predictive generalization capability. In addition, the Adeq Precision values for all models are well above 4, indicating sufficient signal-to-noise ratio within the current design space for reliable process trend analysis and optimization direction prediction.

#### 3.3.4. Response Surface Analysis

**Figure 11:**
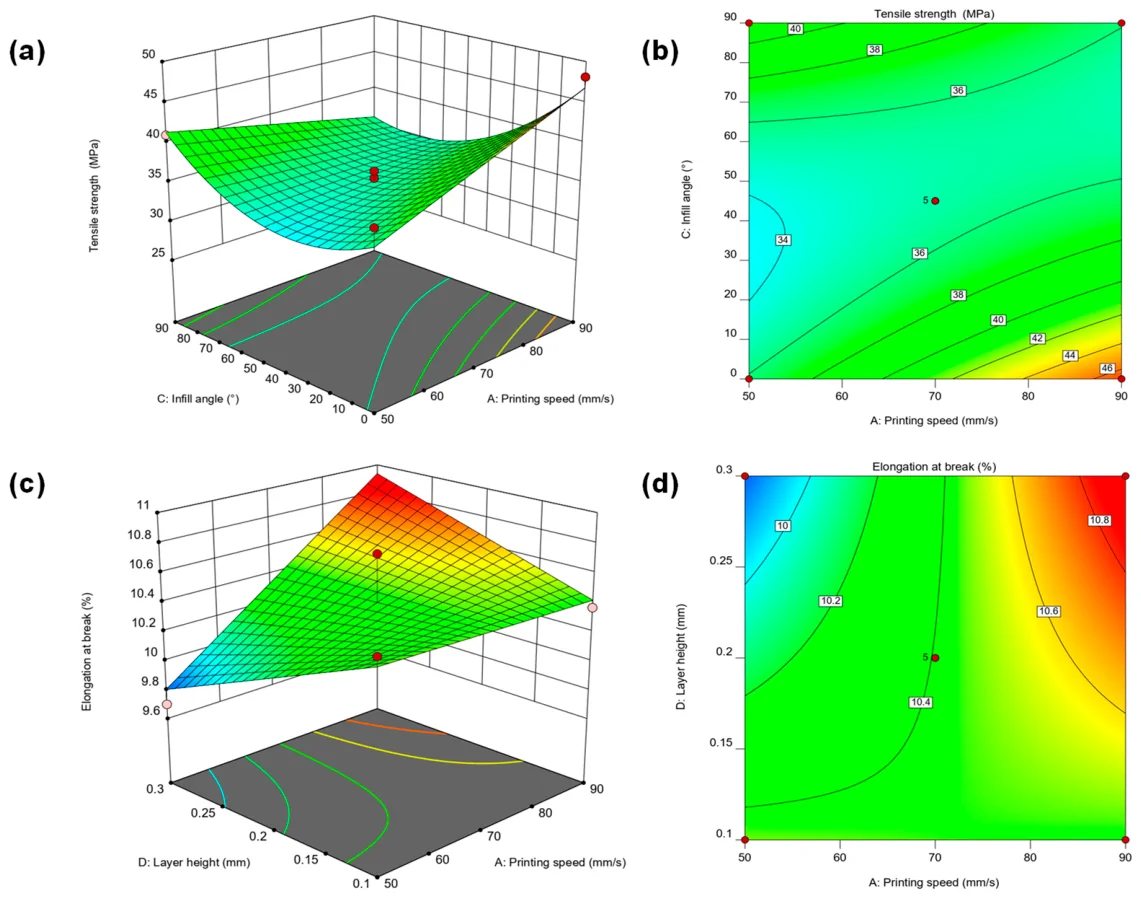
Three-dimensional response surface and contour plots of significant interaction effects: (**a**) 3D response surface plot of *AC* interaction for tensile strength; (**b**) contour plot of *AC* interaction for tensile strength; (**c**) 3D response surface plot of *AD* interaction for elongation at break; (**d**) contour plot of *AD* interaction for elongation at break.

To visualize the significant interaction effects among the factors on the response variables, three-dimensional response surface plots and their corresponding contour plots are presented in Figure 11 for the significant interaction terms identified in Section 3.3.3. The *AC* interaction is shown for tensile strength, and the *AD* interaction for elongation at break. Flexural strength and comprehensive score are excluded from the response surface plots, as no significant interaction effects were detected for these responses.

Figure 11a,b present the interactive effects of *A* and *C* on tensile strength. When the raster angle is fixed, tensile strength generally increases with printing speed, though the magnitude of this response varies with raster angle. At speeds of 60–90 mm/s, tensile strength exhibits a U-shaped trend with raster angle—dual peaks at 0° and 90° and a valley near 45°—while at 50 mm/s, only a single peak at 90° is observed. This indicates that at low speeds, shear-induced orientation is insufficient to activate the 0° axial strengthening mechanism, whereas the 90° crack-deflection toughening effect remains active. The higher strengths at 0° and 90° are observed, with a valley near 45°. The non-parallel contour lines confirm significant *A*–*C* interaction (*p* < 0.05): in the high-angle range (60–90°), tensile strength is less sensitive to speed fluctuations, indicating greater process robustness; in the low-angle range (0–30°), small speed variations cause notable fluctuations, requiring more precise control. The overall peak strength occurs at 90 mm/s and 0°, with a secondary optimum at 50 mm/s and 90°.

Figure 11c,d reveal the coupling between *A* and *D* on elongation at break. Elongation follows an inverted U-shape with speed, peaking at 70–80 mm/s, reflecting the balance between strain hardening and excessive stretching-induced microcracking. Increasing layer height continuously enhances elongation. The curved contours confirm significant *A*–*D* interaction (*p* < 0.05): larger layer heights widen the speed-dependent regulation range, while smaller layer heights yield lower elongation with less speed sensitivity. The optimal toughness window lies at 70–80 mm/s and 0.3 mm layer height.

The optimal parameters for tensile strength and elongation at break are thus in conflict—strength favors high speed (90 mm/s) and axial infill (0°), while elongation favors moderate speed (70–80 mm/s) and large layer height (0.3 mm)—forming a typical Pareto trade-off that underscores the necessity of multi-objective optimization.

#### 3.3.5. Parameter Perturbation Sensitivity Analysis

To quantify the independent effects of individual printing parameters on the mechanical properties and comprehensive score, and to distinguish the contributions of main effects from those of interaction effects, a single-factor perturbation analysis was conducted based on the response surface models. This approach extracts the inherent sensitivity of each factor to the response variables by varying one factor continuously across its range while holding the others at the center point. Its advantage lies in isolating interaction effects and directly revealing the curvature characteristics of the main effects.

**Figure 12:**
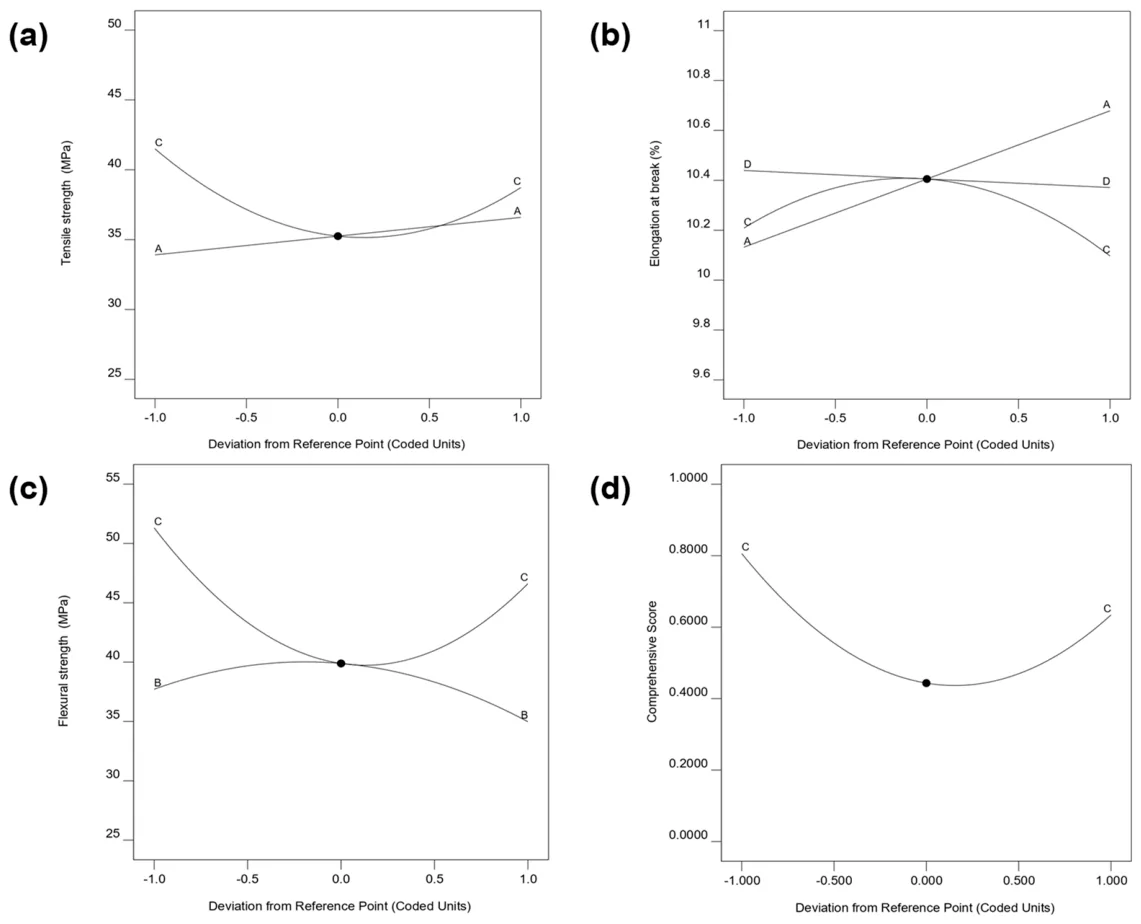
Perturbation plots for (**a**) tensile strength, (**b**) elongation at break, (**c**) flexural strength, and (**d**) comprehensive score. Each curve represents the response variation when one factor is varied across its range while the others are held at the center point.

In conjunction with the response surface analysis in Section 3.3.4, it was found that tensile strength is synergistically regulated by *A* and *C*, while elongation at break is jointly influenced by *A* and *D*, with interaction terms reaching significance in both models (*p* < 0.05). In contrast, for flexural strength and comprehensive score, no significant interaction effects were detected among the factors (*p* > 0.05), and the response behavior is predominantly governed by quadratic main effects. The perturbation curves (Figure 12) further quantify these differences.

Figure 12 presents the single-factor perturbation plots for each response variable. The horizontal axis represents the coded deviation from the center point, and the vertical axis the predicted response. Curve curvature reflects the quadratic effect strength: greater curvature indicates stronger nonlinear influence and higher sensitivity; an approximately straight line indicates linear dominance and limited regulatory capacity.

Figure 12a shows the perturbation characteristics of *A* and *C* on tensile strength. The *C* curve exhibits a pronounced U-shape with a minimum at 45°, while the *A* curve is approximately linear with a gentle slope. The significantly greater steepness of *C* establishes it as the dominant factor, consistent with its highly significant quadratic term (*p* < 0.001), whereas the *A* main effect is non-significant (*p* = 0.134). The distinct morphological difference between the two curves, together with the non-parallel contours, provides visual evidence for the significant *AC* interaction (*p* = 0.014): the influence of *A* on tensile strength depends on *C* level—at 0° axial infill, high speed enhances molecular orientation; at 90° transverse infill, crack deflection dominates and the *A* effect weakens. Thus, optimizing tensile strength should prioritize *C* adjustment, with *A* and *C* configured synergistically.

Figure 12b presents the perturbation curves for elongation at break. *A* shows an approximately monotonic upward trend with the highest sensitivity, serving as the dominant factor (*p* < 0.001). *C* displays a downward-curving parabola, consistent with its significant quadratic term (*p* = 0.006). *D* is nearly horizontal, with negligible influence (*p* = 0.599). The varying curvatures, together with the curved *A*–*D* contours, validate the significant *AD* interaction (*p* = 0.015)—the speed effect is amplified at larger layer height. Optimizing elongation should prioritize *A* as the dominant positive factor, noting that *C* deviation from 45° weakens ductility; *D*’s independent role is limited but larger thickness amplifies the speed benefit, requiring synergistic matching of *A* and *D*.

Figure 12c displays the perturbation curves for flexural strength. Both *A* and *C* are smooth symmetric parabolas with no cross-coupling, consistent with non-significant interaction (*p* > 0.05). *C* shows a minimum at the center with strength increasing toward either side, exhibiting greater curvature and sensitivity (*p* < 0.001). A shows a maximum at the center with strength decreasing toward either side (*p* = 0.033). The two parameters operate independently, allowing stepwise individual adjustment without coupling compensation.

Figure 12d presents the perturbation curves for the comprehensive score. The score is predominantly governed by *C* alone, displaying a standard U-shaped parabola (*p* < 0.001). The remaining three curves are approximately horizontal, with no significant interactions. Process adjustment can effectively improve the comprehensive score through independent optimization of *C*.

Synthesizing the four analyses, the response variables can be classified into two categories according to interaction significance: (1) interaction-dominant type (tensile strength, elongation at break)—characterized by high curvature and mutual interdependencies, requiring synergistic matching of multiple parameters (*A*, *C*, *D*), with *C* being the common factor with the highest sensitivity—and (2) interaction-negligible type (flexural strength, comprehensive score)—characterized by independent parameter effects without complex coupling, allowing stepwise individual adjustment with higher process robustness. All responses tend toward lower values at the center point, and shifting parameters toward either side generally improves performance. Therefore, optimizing tensile strength or elongation at break requires multi-parameter synergy prioritizing *C* in coordination with *A* and *D*; optimizing flexural strength or comprehensive score can be achieved through stepwise adjustment prioritizing *C* without concern for interaction interference.

#### 3.3.6. Determination of Optimal Printing Parameters and Experimental Validation

This section follows a progressive logic—single-objective → multi-objective → entropy-weighted comprehensive score optimization → experimental validation—to hierarchically determine the optimal process parameters and complete model verification. Three sets of single-objective optimizations were first performed using Design-Expert RSM models for tensile strength, elongation at break, and flexural strength. Subsequently, a conventional multi-objective optimization was conducted to balance the three properties. Finally, using the entropy-weighted comprehensive score as the optimization objective, two approaches—“secondary RSM modeling” and “direct BBD experimental ranking”—were employed to identify the global optimum. Parallel mechanical experiments were carried out for all six optimal processes, and predicted values were compared with experimental means to validate model accuracy.

(1) Single-Objective and Subjective Multi-Objective Optimization Results

Using *A*, *B*, *C*, and *D* as optimization variables, four sets of optimal parameters were obtained by maximizing tensile strength, elongation at break, and flexural strength, and equally balancing the three properties, respectively. The corresponding predicted and experimental results are summarized in Table 10.

Table 10 shows good agreement between predicted and experimental values for all optimal schemes, with relative errors within 3%, confirming the predictive reliability of the RSM models for both single-objective and subjective multi-objective optimizations. The low *CV* values across all schemes (0.47–2.63% for *σ_t_*, 0.28–2.37% for *ε_b_*, and 0.42–1.94% for *σ_f_*) further indicate small prediction deviations and high model reliability.

(2) Comparison of two optimization routes based on entropy-weighted comprehensive scores

To explore the engineering value of objective weighting in multi-objective decision-making, two routes were adopted using the entropy-weighted comprehensive score as the optimization objective. Route 1 established an RSM model with the comprehensive scores of each BBD run as a new response variable, followed by continuous optimization in Design-Expert (“entropy weight + DX secondary optimization”). Route 2 directly ranked the BBD experimental data by the measured comprehensive scores (“entropy weight-BBD optimum”).

To ensure fair comparison, the comprehensive scores of all six schemes were uniformly calculated using the same entropy weights and normalization basis as in Section 3.2.3, eliminating evaluation scale differences arising from different optimization objectives. The predicted and experimental comprehensive scores are compared in Table 11.

Table 11 shows that the *CV*s between predicted and experimental comprehensive scores for the three single-objective schemes and the DX multi-objective scheme are 0.53%, 0.76%, 3.32%, and 1.47%, respectively, all below 5%, confirming the high predictive accuracy of the RSM models under single-performance and subjectively equal-weighted conditions.

However, the two entropy-weight-based routes diverge markedly. Both share the same predicted score (0.7706), but the “entropy weight + DX secondary optimization” route yields an experimental score of only 0.7366 (70/200/0/0.2), while the “entropy weight–BBD optimum” route achieves 0.9614 (Run 18, 90/200/0/0.2)—a 30.5% difference and a *CV* of 22.03%. This deviation arises from the nonlinear thermo-mechanical coupling in Run 18, which the low-order RSM treats as residual noise and filters out, resulting in a conservative prediction.

This reveals a general principle: low-order RSM is reliable in gently curved regions but becomes highly uncertain near performance boundaries—Run 18 represents a “local high-performance island” smoothed over by global fitting. Thus, RSM is suitable for broad trend screening, but final decisions should be confirmed experimentally to avoid missing true optima.

In summary, while RSM offers good predictive accuracy under single-objective and subjective multi-objective conditions, secondary RSM modeling and continuous optimization based on the comprehensive score are susceptible to global smoothing, which may direct the search to a mathematically conservative suboptimal region. In contrast, direct ranking of BBD data preserves full physical responses and captures higher-order synergistic gains missed by the model. This demonstrates that in strongly nonlinear additive manufacturing processes, direct ranking of discrete BBD data is superior to model-extrapolated continuous optimization, and physical validation remains the ultimate basis for multi-objective decisions.

(3) Engineering Application Implications

The optimal process identified by entropy weight–BBD direct ranking (90 mm/s, 200 °C, 0° raster angle, 0.2 mm layer height) achieves a comprehensive score of 0.9614, with $\sigma_{t}$ of 48.14 MPa and $\sigma_{f}$ of 51.56 MPa, and small standard deviations ($\sigma_{t}$: ±1.19, $\sigma_{f}$: ±0.79), indicating high repeatability and robustness suitable for batch production.

The 0° raster angle, corresponding to “linear” or “parallel” fill patterns, combined with high speed (90 mm/s) and small layer height (0.2 mm), achieves simultaneous maximization of tensile and flexural strengths under the tested conditions. This suggests that raster angle should not be preset empirically; instead, objective weighting with experimental design should be used for systematic screening.

The “BBD + entropy weight direct ranking” paradigm improves the optimal comprehensive score by 30.5% compared to the conventional “RSM + continuous optimization” strategy (0.7366 → 0.9614). This framework is transferable to other material systems (e.g., ABS, PC, nylon) and multi-objective scenarios (e.g., balancing printing efficiency, surface quality, and material consumption), offering a scientifically grounded tool for managing multi-performance conflicts in engineering practice.

## 4. Discussion

### 4.1. Influence of Blend Ratio on Mechanical Properties and Underlying Mechanisms

The single-factor experimental results demonstrate that the 80:20 PLA/TPU blend ratio achieves the best overall mechanical properties, with tensile strength of 38.93 MPa, elongation at break of 14.12%, and flexural strength of 42.33 MPa—improvements of 39.4%, 15.7%, and 31.6%, respectively, compared to the 70:30 blend. This non-monotonic dependence on TPU content indicates an optimal composition range, beyond which further TPU addition becomes detrimental to load-bearing capacity.

This optimal ratio is consistent with the findings of Rahmatabadi et al. [10], who also identified the 80:20 composition as providing a favorable balance of fracture toughness and tensile properties in PLA/TPU blends. Similarly, Hamidi et al. [14] observed that higher TPU loadings (e.g., 70:30) led to a reduction in tensile and flexural strengths, a trend that aligns with our results.

Multi-scale characterization elucidates the microstructural origins of this composition-dependent performance. FTIR analysis reveals that the 80:20 blend exhibits the most pronounced red shift of the PLA ester carbonyl peak (1750 cm^−1^) and the greatest broadening of the TPU N–H stretching band (3320 cm^−1^), indicating the strongest intermolecular hydrogen-bonding interactions at the phase interface—facilitating efficient stress transfer and suppressing interfacial debonding. XRD confirms that the 80:20 blend retains a crystalline PLA matrix with 20.2% crystallinity, significantly higher than the 70:30 blend (12.3%), where phase inversion occurs and the PLA network integrity is compromised. SEM observations further show that the 80:20 blend exhibits fine, spherical TPU domains uniformly dispersed without significant agglomeration, with fracture surfaces displaying extensive plastic deformation features characteristic of ductile failure. In contrast, the 70:30 blend shows severe phase separation with TPU agglomeration, interfacial debonding, and void formation. Collectively, the strong interfacial hydrogen bonding, the crystalline PLA framework, and uniform elastomeric dispersion in the 80:20 blend are associated with its superior strength–toughness balance. Compared to previous studies [10,14], our multi-scale characterization provides correlated microstructural evidence (FTIR, XRD, SEM) that links the superior performance of the 80:20 blend to specific features—strong interfacial bonding, preserved crystallinity, and uniform phase dispersion—offering a more mechanistic understanding of this composition-dependent behavior.

### 4.2. Feature Dependent Parameter Sensitivity

The ANOVA results reveal a clear hierarchy of parameter importance that varies across different mechanical properties, indicating that tensile strength, elongation at break, and flexural strength are governed by distinct physical mechanisms.

For tensile strength, the response is primarily driven by the *AC* interaction and the quadratic term of *C*^2^, while the main effects of *A* and *C* are not significant (*p* > 0.1). This dominance of nonlinear effects suggests that tensile strength is controlled by the interplay between molecular chain orientation and stress transfer pathways. This observation aligns with Sandanamsamy et al. [33], who identified raster angle as a key factor influencing tensile properties in FDM-printed PLA. The perturbation analysis further reveals that the C curve exhibits a pronounced U-shaped parabolic curvature with a minimum at 45°, while the speed A curve is approximately linear, confirming that *C* is the most influential factor among those tested. The higher tensile strengths at 0° and 90° arise from distinct mechanisms: at 0°, filament alignment maximizes load transfer efficiency [33,34]; at 90°, crack deflection and branching dissipate more fracture energy [35].

For elongation at break, the significant main effect of *A* (*p* < 0.001) and the *AD* interaction (*p* = 0.015), together with the quadratic term of *C*^2^ (*p* = 0.006), indicate a more complex governing mechanism. The positive influence of speed on elongation is attributed to enhanced molecular orientation, imparting strain-hardening capability, while the *AD* interaction reveals that this beneficial effect of speed is modulated by layer height, being more pronounced at larger layer heights, likely due to more complete molecular chain interdiffusion [36]. The response of ductility to layer height appears to be interactive with other parameters, a finding that is qualitatively similar to observations by Sultana et al. [36] on wood–PLA composites, who reported that the effect of layer height on mechanical properties is dependent on the specific parameter combinations.

For flexural strength, the model is mainly governed by the quadratic terms of *B*^2^ (*p* = 0.033) and *C*^2^ (*p* < 0.001), with both main effects non-significant. This indicates that flexural strength is optimized at parameter extremes: high temperature (210 °C) combined with axial (0°) or transverse (90°) infill patterns. The perturbation analysis reveals that both *A* and *C* curves are smooth symmetric parabolas with no cross-coupling, consistent with non-significant interaction (*p* > 0.05), allowing independent adjustment of these parameters.

The comprehensive score model retains only *C*^2^ (*p* < 0.001), with all other effects excluded. While this indicates that raster angle is the sole dominant factor globally, it does not contradict the outstanding performance of Run 18 (90/200/0/0.2) which, despite sharing the same raster angle (0°) with Runs 5 and 7, achieves a significantly higher score (0.9614 vs. 0.7270 and 0.9367). This paradox arises because ANOVA tests global average effects, whereas the nonlinear synergistic gain between speed and layer height at Run 18 occurs locally and does not sufficiently alter the global response surface to reach statistical significance. This reveals the inherent limitation of low-order RSM: statistical non-significance does not imply engineering irrelevance.

Based on these findings, the response variables can be classified into two categories: interaction-dominant (tensile strength, elongation at break), requiring synergistic matching of multiple parameters (*A*, *C*, *D*), and interaction-negligible (flexural strength, comprehensive score), allowing independent parameter adjustment with higher process robustness.

### 4.3. Implications for High-Performance FDM Manufacturing

The optimal process identified through entropy weight–BBD direct ranking (90 mm/s, 200 °C, 0° raster angle, 0.2 mm layer height) achieves an experimental comprehensive score of 0.9614, with tensile and flexural strengths reaching 48.14 MPa and 51.56 MPa, respectively. The small standard deviations ($\sigma_{t}$: ±1.19, $\sigma_{f}$: ±0.79) suggest good repeatability and process robustness, making this parameter set suitable for batch production.

The 0° raster angle, corresponding to “linear” or “parallel” fill patterns in slicing software, combined with high speed (90 mm/s) and moderate layer height (0.2 mm), achieves a favorable balance of high tensile and flexural strengths. This synergistic effect arises from aligned molecular chains along the loading direction, maximizing load transfer efficiency, while moderate layer height ensures sufficient interlayer bonding without excessive voids. Notably, the BBD-optimized speed (90 mm/s) exceeds the single-factor optimum (50 mm/s) for the same blend ratio, reflecting the synergistic interplay between speed, raster angle, and layer height in the multi-objective framework. This higher speed is also less conservative than settings typically recommended for neat PLA systems [6,31], where lower speeds are preferred to enhance interlayer fusion. The difference may be attributed to the broader processing window and lower melt viscosity of the PLA/TPU blend compared to neat PLA, which can facilitate adequate interlayer diffusion even at higher extrusion rates. The difference may be attributed to the broader processing window and lower melt viscosity of the PLA/TPU blend compared to neat PLA, which can facilitate adequate interlayer diffusion even at higher extrusion rates.

The comparison between the two entropy-weight-based decision routes reveals a critical methodological insight. The “entropy weight + DX secondary optimization” route (70/200/0/0.2) yielded an experimental score of only 0.7366, while the “entropy weight–BBD direct ranking” route (Run 18) achieved 0.9614, a 30.5% improvement. This divergence arises because secondary RSM modeling and continuous optimization are susceptible to global smoothing effects, potentially directing the search to a mathematically conservative region. This observation suggests that in highly nonlinear processes such as FDM, reliance solely on RSM-model-recommended optima, a common practice in some optimization studies [24,25], may lead to suboptimal decisions. In contrast, direct ranking of BBD experimental data preserves full physical responses and captures synergistic effects that are not fully described by the quadratic RSM model, a limitation of low-order response surface models that has been noted in the literature [43]. This demonstrates that in strongly nonlinear additive manufacturing processes, direct ranking of discrete BBD data may be more effective than model-extrapolated continuous optimization, and physical validation remains an essential basis for multi-objective decisions.

The “BBD + entropy weight direct ranking” paradigm established in this study is transferable to other additive manufacturing material systems (e.g., ABS, PC, nylon) and multi-objective scenarios (e.g., balancing printing efficiency, surface quality, and material consumption), providing a scientifically grounded and operationally feasible tool for managing multi-performance conflicts.

### 4.4. Limitations and Future Work

Several limitations should be acknowledged. First, the investigation is confined to specific PLA/TPU grades (PLA 2003D and TPU 95A); validation with other grades or polymer blends is needed. Second, mechanical properties were evaluated only through quasi-static tests at room temperature; dynamic properties (impact, fatigue, fracture toughness) and performance under elevated temperatures or cyclic loading were not examined. Third, the low-order RSM approach struggles to capture local nonlinearities, as evidenced by the 22.03% *CV* for Run 18; future work will explore higher-order response surfaces or machine learning-based surrogate models. Fourth, microstructure–property correlations provide indirect evidence; direct in-situ verification (e.g., digital image correlation, real-time SAXS/WAXS) will be incorporated in future studies. Fifth, the entropy weight method does not incorporate subjective preferences; combining it with subjective weighting (e.g., AHP) could tailor optimization to specific applications.

Beyond mechanical performance, the framework could be extended to incorporate printing time, material consumption, surface quality, and energy efficiency as additional objectives. Integrating machine learning with the entropy weight–multi-objective framework could enable adaptive parameter tuning based on real-time sensor feedback, paving the way for closed-loop quality control in FDM.

## 5. Conclusions

This study systematically investigated the optimization of mechanical properties for FDM-printed PLA/TPU blends through an integrated experimental and statistical approach. The main conclusions are as follows:

(1) Optimal blend ratio. In the single-factor experimental stage, the 80:20 PLA/TPU blend ratio was identified as the optimal formulation, achieving tensile strength of 38.93 MPa, elongation at break of 14.12%, and flexural strength of 42.33 MPa—improvements of 39.4%, 15.7%, and 31.6%, respectively, compared to the 70:30 blend. Multi-scale characterization revealed that this superiority originates from strong interfacial hydrogen bonding (FTIR), a well-retained crystalline PLA framework (XRD), and uniform dispersion of fine TPU domains (SEM), collectively achieving an optimum balance between rigid PLA skeletal integrity and flexible TPU toughening efficiency.
(2) Parameter significance. ANOVA results revealed that different mechanical properties are governed by distinct physical mechanisms. Tensile strength is primarily controlled by the *AC* interaction and *C*^2^. Elongation at break is dominated by A and the *AD* interaction. Flexural strength is mainly governed by *B*^2^ and *C*^2^. Raster angle was identified as the most influential factor for the comprehensive score, while speed, temperature, and layer height exhibited minor global main effects.
(3) Optimization strategy. The comparison between two entropy weight-based decision routes revealed that direct ranking of BBD experimental data significantly outperforms secondary RSM modeling followed by continuous optimization. The optimal process parameters identified through BBD direct ranking (90 mm/s, 200 °C, 0°, 0.2 mm) achieved an experimental comprehensive score of 0.9614, representing a 30.5% improvement over the RSM-recommended parameters (70/200/0/0.2, score 0.7366). This demonstrates that in strongly nonlinear FDM processes, physical experimental validation remains the ultimate basis for multi-objective optimization decisions.
(4) Methodological framework. The single-factor experiments identified 80:20 as the optimal blend ratio; on this basis, the BBD response surface method further optimized the printing parameters, establishing 90/200/0/0.2 as the global optimal process parameter combination. The systematic integration of single-factor experiments, BBD, ANOVA, entropy weight-based objective weighting, and experimental validation establishes a robust and reliable framework for multi-objective mechanical property optimization. The optimal parameters (90/200/0/0.2) with small standard deviations ($\sigma_{t}$: ±1.19, $\sigma_{f}$: ±0.79) indicate high process robustness, making this parameter set suitable for batch production of PLA/TPU components requiring balanced mechanical performance.

In summary, this work bridges the gap between material formulation and printing parameter optimization for PLA/TPU blends, offering both mechanistic understanding and engineering guidance for the production of high-performance FDM components.

## Figures and Tables

**Table 1:** Physical and mechanical properties of PLA and TPU pellets.

Property		PLA (NatureWorks 2003D)	TPU (BASF 1195A)
Physical properties	Density (g/cm^3^)	1.24	1.14
	Glass transition temp., Tg (°C)	55–60	−30
	Melting temp., Tm (°C)	140–155	190–210
	Melt flow index (g/10 min)	6 (210 °C/2.16 kg)	_
	Processing temp. range	170–200	170–240
Mechanical properties	Tensile strength (MPa)	60 (yield)	40 (yield)
	Elongation at break (%)	6.0	550
	Elastic modulus (MPa)	3500	—
	Shore hardness	—	96A

Note: All data are sourced from supplier technical datasheets (NatureWorks Ingeo™ 2003D and BASF Elastollan^®^ 1195A).

**Table 2:** Single-Factor Variable Experiment Parameter Settings.

Items	Factor			
	*A* (mm/s)	*B* (°C)	*C* (°)	*D* (mm)
Min	50	190	0	0.1
Max	90	210	90	0.3
Interval	20	10	45	0.1
Fixed value	70	200	45	0.2

**Table 3:** Optimal processing parameters and corresponding mechanical properties for the four PLA/TPU blend ratios determined from single-factor experiments.

Material System	Optimal Process Parameters (*A*/*B*/*C*/*D*)	$\sigma_{t}$ (MPa)	$\varepsilon_{b}$ (%)	$\sigma_{f}$ (MPa)
85:15	50 mm/s;190 °C 0°; 0.1 mm	36.96 ± 0.05	12.50 ± 0.02	40.14 ± 0.04
80:20	50 mm/s;190 °C 0°; 0.1 mm	38.93 ± 0.03	14.12 ± 0.02	42.33 ± 0.03
75:25	90 mm/s;190 °C 0°; 0.1 mm	34.86 ± 0.04	12.40 ± 0.02	31.03 ± 0.03
70:30	70 mm/s; 190 °C 0°; 0.1 mm	27.93 ± 0.03	12.20 ± 0.02	32.15 ± 0.03

Note: $\sigma_{t}$: tensile strength (MPa); $\varepsilon_{b}$: elongation at break (%); $\sigma_{f}$: flexural strength (MPa). Data: mean ± *SD*, *n* = 3. *A*, *B*, *C* and *D* refer to printing speed, nozzle temperature, raster angle and layer height, respectively.

**Table 4:** Factors and levels used in the Box–Behnken design.

Factor	Symbol	Levels		
		−1 (Low)	0 (Center)	+1 (High)
Printing speed (mm/s)	*A*	50	70	90
Nozzle temperature (°C)	*B*	190	200	210
Raster angle (°)	*C*	0	45	90
Layer height (mm)	*D*	0.1	0.2	0.3

**Table 5:** Box–Behnken experimental design and corresponding results.

Run	*A* (mm/s)	*B* (°C)	*C* (°)	*D* (mm)	Mechanical Properties			
					$\sigma_{t}$ (MPa)	$\varepsilon_{b}$ (%)	$\sigma_{f}$ (MPa)	Y
1	−1	−1	0	0	30.43 ± 1.25	9.85 ± 0.33	33.70 ± 1.08	0.1395
2	+1	−1	0	0	37.22 ± 1.81	10.82 ± 0.13	35.96 ± 1.22	0.4485
3	−1	+1	0	0	35.04 ± 0.71	10.32 ± 0.08	42.77 ± 2.12	0.4991
4	+1	+1	0	0	37.89 ± 1.09	10.66 ± 0.42	30.65 ± 1.21	0.3353
5	0	0	−1	−1	39.49 ± 0.31	9.87 ± 0.18	49.63 ± 2.05	0.7270
6	0	0	+1	−1	41.16 ± 1.04	9.90 ± 0.42	47.97 ± 1.48	0.7362
7	0	0	−1	+1	44.26 ± 0.49	10.49 ± 0.24	51.12 ± 1.65	0.9367
8	0	0	+1	+1	41.01 ± 0.40	10.20 ± 0.27	50.41 ± 1.70	0.8118
9	−1	0	0	−1	32.14 ± 0.83	10.52 ± 0.52	35.64 ± 0.89	0.2840
10	+1	0	0	−1	38.30 ± 0.60	10.37 ± 0.30	44.20 ± 1.86	0.6193
11	−1	0	0	+1	36.86 ± 0.85	9.70 ± 0.18	39.01 ± 1.66	0.4103
12	+1	0	0	+1	33.51 ± 1.12	10.72 ± 0.20	37.92 ± 1.67	0.3869
13	0	−1	−1	0	36.91 ± 1.35	10.40 ± 0.47	51.47 ± 1.61	0.7459
14	0	+1	−1	0	41.81 ± 0.23	10.21 ± 0.23	50.01 ± 1.74	0.8246
15	0	−1	+1	0	38.51 ± 0.06	10.29 ± 0.27	47.71 ± 0.54	0.6952
16	0	+1	+1	0	35.77 ± 0.43	10.13 ± 0.24	36.93 ± 0.91	0.3731
17	−1	0	−1	0	38.39 ± 0.24	9.88 ± 0.24	47.00 ± 1.92	0.6413
18	+1	0	−1	0	48.14 ± 0.93	10.40 ± 0.52	51.56 ± 1.86	0.9614
19	−1	0	+1	0	41.01 ± 1.02	9.74 ± 0.20	47.09 ± 1.89	0.6992
20	+1	0	+1	0	34.91 ± 0.46	10.32 ± 0.01	42.48 ± 1.28	0.4893
21	0	−1	0	−1	42.67 ± 1.12	10.74 ± 0.44	45.74 ± 1.22	0.7985
22	0	+1	0	−1	37.69 ± 0.70	10.85 ± 0.05	35.47 ± 1.61	0.4525
23	0	−1	0	+1	28.81 ± 0.72	10.40 ± 0.21	29.86 ± 0.89	0.600
24	0	+1	0	+1	35.81 ± 1.23	10.33 ± 0.27	32.18 ± 1.09	0.2868
25	0	0	0	0	35.59 ± 0.78	10.17 ± 0.12	42.51 ± 1.01	0.4947
26	0	0	0	0	34.06 ± 1.38	10.25 ± 0.23	39.42 ± 1.56	0.3938
27	0	0	0	0	32.45 ± 0.88	10.13 ± 0.15	41.67 ± 1.03	0.3913
28	0	0	0	0	34.30 ± 0.75	10.73 ± 0.35	39.43 ± 0.25	0.4414
29	0	0	0	0	36.50 ± 0.72	10.33 ± 0.26	43.66 ± 0.31	0.5573

**Table 6:** ANOVA results for the tensile strength response surface model.

Source of Variation	Sum of Squares	Degree of Freedom	Mean Square	F-Value	*p*-Value	Partial *η*^2^
Model **	273.41	4	68.45	7.63	<0.001	
*A*	21.60	1	21.60	2.41	0.134	0.091
*C*	23.05	1	23.05	2.57	0.122	0.097
*AC* *	62.81	1	62.81	7.00	0.014	0.226
*C*^2^ **	166.36	1	166.36	18.55	<0.001	0.436
Residual	215.24	24	8.97			
Lack of Fit	205.64	20	10.28	4.29	0.084	
Pure Error	9.59	4	2.40			
Cor Total	489.05	28				

Note: ** *p* < 0.01, * *p* < 0.05. Partial *η*^2^ represents the partial eta-squared effect size, indicating the proportion of variance in the response variable explained by each effect, $R^{2}=0.5599;\;\;R_{\mathrm{Adj}}^{2}=0.4865;\;R_{\mathrm{Pred}}^{2}=0.4031;\;\mathrm{Adeq}\;\mathrm{Precision}=10.3689$.

**Table 7:** ANOVA results for the elongation at break response surface model.

Source of Variation	Sum of Squares	Degree of Freedom	Mean Square	F-Value	*p*-Value	Partial *η*^2^
Model **	1.74	5	0.35	7.07	<0.001	
*A* **	0.90	1	0.90	18.21	<0.001	0.443
*C*	0.04	1	0.04	0.76	0.392	0.034
*D*	0.01	1	0.01	0.28	0.599	0.012
*AD* *	0.34	1	0.34	6.95	0.015	0.231
*C*^2^ **	0.45	1	0.45	9.13	0.006	0.285
Residual	1.13	23	0.05			
Lack of Fit	0.90	19	0.05	0.82	0.665	
Pure Error	0.23	4	0.06			
Cor Total	2.87	28				

Note: ** *p* < 0.01, * *p* < 0.05. Partial *η*^2^ represents the partial eta-squared effect size, indicating the proportion of variance in the response variable explained by each effect, $R^{2}=0.6057;\;\;R_{\mathrm{Adj}}^{2}=0.5200;\;R_{\mathrm{Pred}}^{2}=0.3842;\;\mathrm{Adeq}\;\mathrm{Precision}=11.2122$.

**Table 8:** ANOVA results for the flexural strength response surface model.

Source of Variation	Sum of Squares	Degree of Freedom	Mean Square	F-Value	*p*-Value	Partial *η*^2^
Model **	818.19	4	204.55	12.1	<0.001	
*B*	22.5	1	22.5	1.33	0.260	0.053
*C*	66.27	1	66.27	3.92	0.059	0.140
*B*^2^ *	86.69	1	86.69	5.13	0.033	0.176
*C*^2^ **	568.08	1	568.08	33.6	<0.001	0.583
Residual	405.82	24	16.91			
Lack of Fit	391.63	20	19.58	5.52	0.055	
Pure Error	14.19	4	3.55			
Cor Total	1224.01	28				

Note: ** *p* < 0.01, * *p* < 0.05. Partial *η*^2^ represents the partial eta-squared effect size, indicating the proportion of variance in the response variable explained by each effect, $R^{2}=0.6684;\;\;R_{\mathrm{Adj}}^{2}=0.6132;\;R_{\mathrm{Pred}}^{2}=0.4923;\;\mathrm{Adeq}\;\mathrm{Precision}=9.5671$.

**Table 9:** ANOVA results for the comprehensive score response surface model.

Source of Variation	Sum of Squares	Degree of Freedom	Mean Square	F-Value	*p*-Value	Partial *η*^2^
Model **	0.63	2	0.31	14.02	<0.001	
*C*	0.09	1	0.09	3.97	0.057	0.134
*C*^2^ **	0.54	1	0.54	24.08	<0.001	0.482
Residual	0.58	26	0.02			
Lack of Fit	0.56	22	0.03	5.10	0.062	
Pure Error	0.02	4	0.01			
Cor Total	1.21	28				

Note: ** *p* < 0.01. Partial *η*^2^ represents the partial eta-squared effect size, indicating the proportion of variance in the response variable explained by each effect, $R^{2}=0.5189;\;\;R_{\mathrm{Adj}}^{2}=0.4819;\;R_{\mathrm{Pred}}^{2}=0.4009;\;\mathrm{Adeq}\;\mathrm{Precision}=7.5402$.

**Table 10:** Single-objective and subjective multi-objective optimal process parameters with experimental validation.

Objective	Optimal Parameters *A*/*B*/*C*/*D*	Predicted			Experimental			*CV* (%)		
		$\sigma_{t}$ (MPa)	$\varepsilon_{b}$ (%)	$\sigma_{f}$ (MPa)	$\sigma_{t}$ (MPa)	$\varepsilon_{b}$ (%)	$\sigma_{f}$ (MPa)	$\sigma_{t}$	$\varepsilon_{b}$	$\sigma_{f}$
*σ_t_*	90/200 /0/0.2	46.80	10.48	51.33	47.59 ± 1.19	10.55 ± 0.12	49.94 ± 0.35	1.18	0.47	1.94
*ε_b_*	90/205.82/50.49/0.28	48.14	10.85	37.74	46.75 ± 0.46	11.22 ± 0.24	38.19 ± 1.28	2.07	2.37	0.84
*σ_f_*	60.38/198.07/0/0.16	38.95	10.14	51.45	37.53 ± 1.00	10.18 ± 0.28	51.76 ± 0.79	2.63	0.28	0.42
DX multi- objective	90/198.67/0/0.3	46.80	10.74	51.43	46.79 ± 1.13	10.72 ± 0.11	50.85 ± 0.96	1.48	1.01	0.80

Note: *CV* is the coefficient of variation between predicted and experimental values: *CV* = |*Predicted* − *Experimental*| / ((*Predicted* + *Experimental*)/2) × 100%. DX multi-objective denotes equal-weight (1/3 each) multi-objective optimization using Design-Expert software.

**Table 11:** Comparison of predicted and experimental comprehensive scores for different optimization strategies.

Optimization Strategy	Optimal Parameters	Predicted Score	Experimental Score	*CV* (%)	Rank
Entropy weight + DX secondary optimization (non-BBD)	70/200/0/0.2	0.7706	0.7366	4.51	4
Entropy weight–BBD optimum (Run 18)	90/200/0/0.2	0.7706	0.9614	22.03	1
Tensile strength optimum	90/200/0/0.2	0.9449	0.9399	0.53	3
Elongation at break optimum	90/205.82/50.49/0.28	0.7174	0.7229	0.76	5
Flexural strength optimum	60.38/198.07/0/0.16	0.7340	0.7100	3.32	6
DX multi-objective optimization	90/198.67/0/0.3	0.9674	0.9533	1.47	2

Note: ① Ranking is based on experimental comprehensive score (descending order); ② *CV* is calculated as the coefficient of variation between predicted and experimental values, following the same formula as in Table 10; ③ the two entropy weight-based strategies share the same predicted score (0.7706) but yield experimental scores of 0.7366 and 0.9614, respectively, with a 30.5% difference between them; ④ although the “tensile strength optimum” and the “entropy weight-BBD optimum (Run 18)” share the same process parameters (90/200/0/0.2), their comprehensive scores are derived from different sources—the former was retrospectively evaluated using the entropy weights after single-objective optimization, while the latter was directly selected by ranking the measured comprehensive scores in the BBD dataset (see Section 3.3.6 for details).

## Data Availability

The raw data supporting the conclusions of this article will be made available by the authors on request. The data are not publicly available due to ongoing collaborative research.

## Abbreviations

The following abbreviations are used in this manuscript:

PLA	Polylactic Acid
TPU	Thermoplastic Polyurethane
FDM	Fused Deposition Modeling
FTIR	Fourier Transform Infrared
XRD	X-ray Diffraction
SEM	Scanning Electron Microscopy
ANOVA	Analysis of Variance
BBD	Box–Behnken Design
*σ_t_*	Tensile Strength
*ε_b_*	Elongation at Break
*σ_f_*	Flexural Strength
*Y*	Comprehensive Score
